# Keep on truckin’: how effective are health behaviour interventions on truck drivers’ health? A systematic review and meta-analysis

**DOI:** 10.1186/s12889-024-19929-1

**Published:** 2024-09-27

**Authors:** Rosa Virgara, Ben Singh, Edward O’Connor, Kimberley Szeto, Zydan Merkx, Christian Rees, Nicholas Gilson, Carol Maher

**Affiliations:** 1 Allied Health and Human Performance, University of South Australia, c/o GPO Box 2471, Adelaide, South Australia 5001 Australia; 2 Health and Biosecurity, Commonwealth Scientific and Industrial Research Organisation, Adelaide, South Australia Australia; 3 School of Human Movement and Nutrition Sciences, The University of Queensland, Brisbane, Queensland Australia

**Keywords:** Physical activity, Diet, Cardiometabolic health biomarkers, Freight worker

## Abstract

**Background:**

Truck drivers are a vital workforce, but have higher rates of obesity and other chronic diseases than the general population. The occupation’s sedentary nature, limited physical activity opportunities and access to healthy food, and irregular sleeping patterns contribute to poor health. This systematic review and meta-analysis aimed to evaluate the effectiveness of interventions on health behaviours and cardiometabolic biomarkers of health in truck drivers.

**Methods:**

A systematic search was conducted in February 2024, and reported according to PRISMA 2020 guidelines. Experimental studies targeting physical activity, sedentary behaviour, sleep, diet, weight loss, drug/alcohol use, and/or smoking were eligible. Two reviewers independently screened and completed data extraction and risk of bias assessment. Data were combined at the study level. Pooled statistics were calculated using mean differences (MD) or standardised mean differences (SMD) for outcomes that were reported in ≥2 studies. Pre- and post-intervention means and standard deviations (SD) for the intervention and control groups were used to compute effect sizes.

**Results:**

Nineteen studies (*n*=2137 participants) were included. Meta-analyses found a small-to-moderate increase in fruit and vegetable consumption (SMD 0.32, *p*=0.03) with no other significant effects on other outcome variables.

**Conclusions:**

Interventions are moderately effective in increasing truck drivers’ fruit and vegetable consumption, but not other outcomes. There is a dearth of research in the driver population compared to other occupational groups. Future interventions should consider workplace and environmental factors to promote the health and wellbeing of truck drivers.

**Trial registration:**

The study protocol was registered on PROSPERO (CRD42021283423).

**Supplementary Information:**

The online version contains supplementary material available at 10.1186/s12889-024-19929-1.

## Background

### Rationale

Truck drivers provide a vital service to global economies. In the United States (US), it is estimated that 71.6%, or $10.4 trillion, of the total value of commodities [1] shipped annually is transported by trucks. In Australia, this was $224.2 billion [2]; whilst in the United Kingdom (UK) truck transportation accounts for 98% of all consumer products and machinery being transported by freight, valued at £124 billion per year [3]. Not only is truck transportation a billion-dollar industry, but it also employs up to 5.8% of the total US workforce [4], with similarly high rates of employment observed in the UK [3] and Australia [2].

Truck driving is characterised by irregular shift work, prolonged working hours while driving, sleep deprivation [5], and limited access to healthy food [6]. The health and safety risks associated with shift work are well established [7], and the occupational demands and environment of truck driving present limited opportunities for healthy behaviour. Consequently, unhealthy lifestyle behaviours including poor dietary choices, high amounts of physical inactivity and sedentary behaviour, smoking, excessive alcohol consumption and sleep deprivation are highly prevalent amongst truck drivers [8, 9]. Moreover, many truck drivers also have poor cardiometabolic risk profiles, including high rates of overweight and obesity, hypertension, hypercholesterolaemia, and elevated blood glucose [8]. More than half (54.3%) of truck drivers in Australia are obese, compared to the national obesity rate of 32.5% [10]. The truck driving profession is also ranked as having one of the highest rates of illness and occupational injuries such as depression and back pain [11]. Despite the high prevalence of poor health behaviours and the disproportionate impact of chronic disease risk factors, truck drivers have limited access to public and private healthcare and social support networks [12]. Furthermore, drivers also tend not to access healthcare when injury or illness occurs, which can allow acute health issues to become chronic [13].

Effective health promotion programs and interventions targeting truck driver health behaviours are critical to addressing the substantial health risks and chronic disease prevalence in this population. A 2015 systematic review with narrative synthesis [14] investigating health promotion interventions for truck drivers identified some minor, short-term improvements in health behaviours (diet, physical activity (PA) and health outcomes (Body Mass Index (BMI), percentage of body fat, and blood pressure). However, the review had several methodological limitations, such as article screening and data extraction not performed in duplicate. Moreover, outcomes were descriptively synthesised based on direction of effect and statistical significance within each individual study, with no meta-analysis completed. Since this first systematic review, two other relevant reviews have been conducted [8, 15]. One [8] narratively synthesised data describing truck drivers’ health and risk behaviours, cardiometabolic health biomarkers, and mental health, but did not evaluate effectiveness of interventions. The other [15] focused on the effectiveness weight loss interventions for truck drivers. This review concluded that interventions may support successful weight loss, but there were few studies (*n*=5), low certainty of evidence, and results were synthesised via narrative synthesis only.”

### Objectives

Given these limitations there is a need to update and synthesise the current evidence base, including recent intervention studies to improve truck drivers’ health outcomes [16–18], using current best practice systematic review methodologies. Further, meta-analyses are required to inform a more precise estimate of intervention effects on relevant health-related outcomes. This will inform the future planning of interventions and programs to improve truck drivers’ health. Therefore, this study aimed to conduct a systematic review with meta-analysis to evaluate the effectiveness of interventions on health behaviours and cardiometabolic health biomarkers in truck drivers.

## Methods

This systematic review is reported in accordance with the Preferred Reporting Items for Systematic Reviews and Meta-Analyses statement [19].

### Eligibility criteria

We included studies investigating the effects of interventions on health behaviours and cardiometabolic health biomarkers in truck drivers. Eligible study designs were experimental studies including, randomised controlled trials (RCTs), cluster RCTs, quasi-experimental studies and pre-post studies. Where samples included various types of drivers (e.g., truck, taxi, bus), ≥ 50% of the sample had to be identified as “truck” drivers to be included. We included studies of health behaviour interventions that targeted one or more of the following: PA, sedentary behaviour, sleep, diet, weight loss, drug use, alcohol use, and smoking. Interventions that did not target cardiometabolic health outcomes were not included (e.g., pain). We did not impose restrictions based on the number of participants, or intervention delivery format, duration, or frequency.

### Information sources and search strategy

We searched MEDLINE, Embase and Emcare electronic databases from inception to February 2024. Search strategies were developed with guidance from an academic librarian and employed keywords and MeSH subheadings related to truck drivers and motor vehicles, and health behaviour interventions (e.g., exercise, diet, tobacco, sleep, alcohol). The complete search strategy is available in Supplementary File 1. We searched key journals and screened reference lists of included studies to identify additional eligible studies. Grey literature was not included in the search.

### Selection process

Identified studies were imported to Endnote [20], where duplicates were removed, and then imported to Covidence [21] where screening was carried out using a criteria checklist (Supplementary File 2). Screening was conducted in two stages: 1) titles and abstracts, and 2) full text. Studies were screened in duplicate at each stage by five reviewers (RV, KS, EO, CR, and ZM). All disagreements were resolved by discussion.

### Data collection process, data items and study risk of bias assessment

Three reviewers (RV, KS, EO) independently extracted data and assessed study quality in duplicate using a customised data extraction form (Supplementary File 3). Extracted data were compared for consistency, with discrepancies resolved through discussion between reviewers. We extracted data related to study characteristics (study design, country, sampling strategy, sample size), participant characteristics (age, sex, ethnicity), intervention characteristics (health behaviours targeted, duration, use of theory), outcome measures (type of measure, tool used, validity/reliability, measurement timepoints), and results (as means and standard deviations, *p*-values). We used the Critical Appraisal Skills Program (CASP) [22] for RCTs to assess risk of bias. Attention was given to the domains: randomisation processes, blinding of participants and reporting of results. For non-randomised trials, items related to randomisation processes and participant blinding were scored as “Not applicable”.

### Effect measures and synthesis methods

The outcomes of interest were PA, sedentary behaviour, sleep, diet, smoking, and alcohol consumption and cardiometabolic health biomarkers, including BMI, weight, waist circumference, blood pressure, and blood cholesterol.

Data were combined at the study level. Pooled statistics were calculated using mean differences (MD) or standardised mean differences (SMD) using RevMan software [23] for outcomes that were reported in ≥ 2 studies. Pre- and post-intervention means and standard deviations (SD) for the intervention and control groups were used to compute effect sizes. If means and SD were not reported, authors were contacted or mean and/or SD were estimated from reported data (e.g., median and range) using recommended formulas [24]. Studies were excluded from the meta-analyses if the authors could not be contacted and mean or SD could not be estimated.

Meta-analyses for each outcome were undertaken to 1) compare the effects of intervention versus comparison groups (using post-intervention means and SD of the intervention and comparison groups from RCTs only), and 2) assess the change in outcomes between pre-and post-intervention (using pre- and post-intervention data from all included studies). Heterogeneity was assessed using the I^2^ statistic to quantify the proportion of total variability in study estimates attributable to heterogeneity rather than sampling error [24]. The following cut-offs for I^2^ were used: 0–29%= no heterogeneity; 30–49%= moderate heterogeneity; 50–74%= substantial heterogeneity; and 75–100%= considerable heterogeneity [25]. Publication bias was assessed using funnel plots [26]. SMDs and corresponding standard errors were plotted against each other, and asymmetries or missing sections within the funnel plot were assessed to determine the presence of publication bias [26]. Standardised classifications for the magnitude of effect were used: 0.20 representing a small effect, 0.50 representing a medium effect, and 0.80 representing a large effect [27].

### Reporting bias and certainty assessment

Certainty of evidence for each outcome was assessed by RV using the GRADE approach [28]. Certainty of evidence for individual outcomes were graded as follows:

- High certainty: further research is very unlikely to change our confidence in the effect estimate.
- Moderate certainty: further research is likely to have an important impact on our confidence in the effect estimate and may change the estimate.
- Low certainty: further research is very likely to have an important impact on our confidence in the effect estimate and may change the estimate.
- Very low certainty: we are very uncertain about the effect estimate.

We exported data from RevMan5 [23] into GRADEpro GDT software [29] to produce a summary of findings table for; assessment tools, follow‐up range, timing of follow‐up, study design, number of studies, total sample sizes, effect estimates and certainty of the evidence. The table was generated based on the recommendations of the Cochrane Handbook for Systematic Reviews of Interventions [30] and included; primary and secondary outcomes in the review, intervention effects; the number of participants and studies addressing each outcome and; a grade for the overall certainty of the body of evidence for each outcome.

## Results

### Study selection

**Fig. 1:**
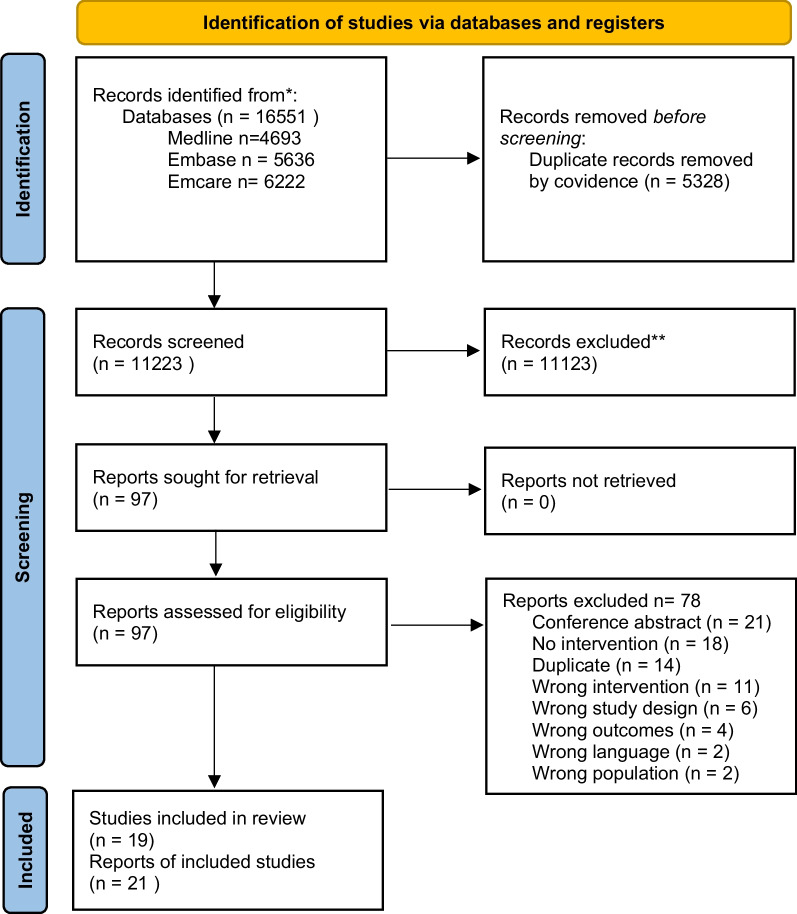
PRSIMA Flow diagram

The electronic search yielded 1122 results after 5328 duplicates were removed. No additional records were identified from hand-searching key journals or reviewing the reference lists of included studies. A total of 96 full texts were reviewed, from which 18 studies (21 reports) were included in the review (see Fig. 1 for reasons for exclusion, supplementary file 4 for individual studies excluded). One further study was identified from contacting authors, to provide a total of *n*=19 included studies. In three instances, outcomes from a single sample were presented across three studies (Gilson et al., 2016 [31], 2017 [16]; Puhkala et al., 2015 [32], 2016 [33] and Clemes et al. 2022 [34], Ruettger et al. 2022 [35] and Guest et al. 2023 [36]. This review presents findings from each of these publications as a single study.

### Study characteristics

A summary of study characteristics is shown in Table 1: Overview of Included Studies. Of the 19 included studies (23 individual papers), k=3 were RCTs [32, 33, 37, 38] k= 3 were quasi-RCTs [39–41], k=4 were cluster RCT [18, 34–36, 42, 43], k=7 were single-arm pre-post [16, 17, 31, 44–49] , and k=1 was a pilot within-subjects study [50]. Studies were conducted in the US (k=9), Europe (k=2 Finland, k=2 United Kingdom, k=1 Sweden, k=1 Germany) and Australia (k=3) and Taiwan (k=1). Interventions were conducted at worksites/truck depots in k=16 studies, and the intervention setting was unclear in k=3 studies [32, 33, 39, 40].

**Table 1:** Overview of included studies

**Study details**	**Sample characteristics**	**Health Behaviours Targeted**	**Outcome Measure: Assessment Method**	**Intervention Components**
**Author** Olson et al. 2023 [42] **Country** United States **Design** Cluster RCT	Total *n*=49 Intervention group *n*= 25 mean age 40.2 (11.05) years Control Group *n*= 24 mean age 41.7 (14.55) years Male participants: 81.25%	PA Diet Weight loss Sleep	**Objective tools** • IPAQ • Actigraph (GT3x) • Body composition (weight, height, BMI) **Self-Report** • Sleep quality • Sleep quantity • PROMIS sleep disturbance Sleep hygiene index	**Summary** Participants received a combination of cab enhancements followed by a behavioural program. The cab enhancements included drivers seats that reduced vibrations and mattresses to support different body regions. The behavioural program focussed on increasing PA and improving sleep hygiene. It was delivered via an online web application with an inter-team walking competition including individual and team goal setting, self-monitoring and feedback, online training and calls with a health coach. **Duration** 3 months **Follow up from completion** Nil **Theory** Not stated
**Author** Gilson et al. 2023 [43] **Country** Australia **Design** Cluster RCT	Total *n*=44 Intervention group *n*= 27 Control Group *n*= 17 Entire sample Mean Age 50.5 (9.8) years Male participants: 100%	PA	**Objective tools** • Actigraph (GT3x) • Cardiorespiratory Fitness (Vo2 Max) • Cardiopulmonary Exercise Test (CPET) • Body composition (weight, height, BMI) **Self-Report** • Physical activity • Fruit and Veg intake	**Summary** A 12 week, high intensity interval training physical activity intervention using both cycle ergometry and body weight exercises. A stepped-down model was used whereby sessions were initially completed at work under supervision of an exercise physiologist and gradually phased out to self-managed participation. Participants encouraged to complete a minimum of three HIIT sessions per week, with each HIIT session of a 4 minute duration **Duration** 3 months **Follow up from completion** Nil **Theory** None – used the evidence of the effectiveness of High Intensity Interval Training
**Author** Clemes et al. 2022 [34] **Country** United Kingdom **Design** Cluster RCT	Total *n*=382 Intervention group *n*= 183 mean age 48.6 (9.1) years Control Group *n*= 199 mean age 48.3 (9.7) years Male participants: 99%	Physical activity Diet	**Objective tools** • Accelerometry (activPAL) • GENEactiv • Body composition (weight, height, BMI) **Self-Report** Nil	**Summary** A group-based education session designed to support drivers to acquire knowledge about the links between PA, diet and sitting and type 2 diabetes and CVD risk. Education sessions also involved discussion of feasible strategies to improve health behaviours during work and non-work hours, activities to gain knowledge on nutritional composition of different foods and drinks, as well as individualised goal setting and action planning. Intervention group received a Fitbit to monitor daily step counts (with real time feedback available), a cab workout and exercise equipment to complete the workout. The session was co-delivered by research team members and an individual champion from each site (i.e., a driver who was trained to facilitate the session, and who also acted as an ongoing health coach support to the other drivers). Additional text message support was provided by the research team throughout the 6-month intervention. **Duration** 6 months **Follow up from completion** 16-18 months **Theory** Social cognitive theory
**Author** Chang et al. 2022 [39] **Country** Taiwan **Design** Quasi-RCT	Total *n*= 242 Intervention group *n*=125 mean age 47.8 (5.41) years Control Group *n*= 117 mean age 47.4 (7.77) years no details on gender	Diet	**Objective tools** • Body composition (weight, height, BMI) **Self-Report** • Food Frequency Scale • Taiwan national health interview • Self-perceived susceptibility • Self-perceived severity • Self-efficacy • Perceived barriers to cues to action	**Summary** The purpose of this program was to improve participants’ awareness of disease risks, improve their self-efficacy and perceived benefits, reduce their perceived barriers, and promote changes in their healthy eating behaviours by increasing their exposure to cues to action. The intervention program involved online messages (the five concepts (i.e., perceived susceptibility, perceived severity, cues to action, perceived barriers, and self efficacy) of the HBM as well as simple techniques for implementing healthy eating), online instant responses from a dietitian or nurse, a picture-based food log, an audio e-book, and a loyalty e-card. The loyalty card encouraged drivers to upload photos of their healthy meals for points. **Duration** 12 weeks **Follow up from completion** Nil **Theory** Health Belief Model
**Author** Gawlik et al. 2022 [37] **Country** Germany **Design** RCT	Total *n*= 140 Intervention group 1 *n*= 50 mean age 47.3 (10.0) years Intervention group 2 *n*= 61 mean age 49 (10.4) years Control Group *n*= 29 mean age 46.5 (9.8) years Male participants: 93.7%	Physical Activity	**Objective tools** • Nil **Self-Report** • Workplace physical activity questionnaire • Behaviour regulation in sport questionnaire 24 • Multidimension exercise self-efficacy	**Summary** Both intervention group 1 and intervention group received motivational coaching, whilst group 2 received additional volitional coaching in addition to motivational coaching. The control group (CG) received no coaching. In addition, participants in each group were provided a vehicle-integrated fitness device. This is a fitness device for performing physical exercises in the driver’s cab, developed by the multinational automotive corporation. Both coaching comprised two sessions with an interval of four weeks. All coaching sessions took place over the telephone. The duration was approximately 15 min per call. Motivational coaching aimed at improving the truck drivers’ behavioural internalization. In the motivational coaching sessions, coach and coachee (coached truck driver) talked about the personal motivation for study participation and for exercising with the vehicle-integrated fitness device. In addition, they exchanged about positive and negative outcome expectancies and set a physical activity goal. **Duration** 20 weeks **Follow up from completion** 4 weeks **Theory** Self-efficacy theory (Bundarra implementation intention technique)
**Author** Pylkkonen et. al 2018 [38] **Country** Finland **Design** RCT	Total *n*=53 Intervention group *n*=32 intervention group, mean age 38.2 (11.3) years Control Group *n*=21, mean age 37.9 (9.4) years Male participants: not provided	Sleep	**Objective tools** • Actigraphy **Self-Report** • Karolinska Sleepiness Scale • Subjective sleepiness • use of sleep counterness measures	**Summary** Educational training session for 3 hours 30 minutes with 45 minute lecture and 1 hour workshop to complete written exercises on improving sleep. In addition participants given personal advice on how to counteract sleepiness, optimal sleep, napping, caffeine use, physical activity and nutrition. **Duration** 8 weeks **Follow up from completion** Unclear in published paper - post measures taken 4-5 months post intervention. Average duration between pre and post = 362 days. **Theory** Transtheoretical model
**Author** Olson et al. 2016 [18] **Country** United States **Design** Cluster RCT	Total *n*=472 Intervention group *n*=229, mean age 47.9 (11.2) years, Male participants: 87% Control group *n*=223, mean age 47.6 (11.6) years Male participants: 86%	Weight loss Physical activity Diet Sleep	**Objective tools** • Body composition (weight, height, BMI, waist circumference, % body fat) • Blood pressure • Fasting bloods (lipids, glucose) **Self-Report** • Healthy Physical activity Scale • Dietary intake • Pittsburgh Sleep Quality Index	**Summary** Participants set a weight loss goal, worked in groups of 10-18 people, group with highest percentage weight loss were rewarded with merchandise and $100 vouchers. Drivers had to log weight and days/week meeting behavioural goals related to physical activity, nutrition, sleep. During the intervention groups were given feedback. **Duration** 6 months **Follow up from completion** Nil **Theory** none stated
**Author** Puhkala 2016 [33] 2015 [32]^$^ **Country** Finland **Design** RCT	Total *n*=113 Intervention group *n*=55, mean age 47.6 (7.9) years Control group *n*=58, mean age 46.5 (8.6) years, Male participants: 100%	Physical Activity^$^ Diet^$^ Weight loss Sleep^$^	**Objective tools** • Pedometer • Body Composition (weight, height, % fat, circumference) • Blood pressure • Blood samples ( fasting Glucose and Cholesterol) **Self-Report** • Modified International Physical Activity Questionnaire • Food diary	**Summary** Individual Lifestyle counselling - 6 x 60 minute face to face counselling sessions and 7 x 30 min phone contacts with trained counsellors. Counselling focused on supporting participant to set and strive toward dietary targets (e.g., serves of fruit and veg; improved fat quality, reduced low-quality CHOs) and PA targets[1, 2] (increase step count by 4000 steps daily). **Duration** 12 months **Follow up from completion** 24 months **Theory** Health Action Process Approach
**Author** Hedberg et al. 1998 [40] **Country** Sweden **Design** Quasi-RCT	Total *n*= 97 Intervention Group: *n*=49, mean age 42.9 years; Control Group: *n*=48, mean age 43.4 years) Male participants: 100%	Physical activity Diet Smoking cessation	**Objective tools** • Body composition (weight, height, bmi, • Blood pressure • Fasting bloods (lipids, glucose) • Maximal oxygen uptake based on HR **Self-Report** Questionnaires on lifestyle habits (exercise, diet, smoking) but specific questionnaire not provided	**Summary** Individualised advice provided on changes to physical activity, diet and smoking behaviours based on responses to questions from baseline questionnaire. There was a focus on current behaviour and desired behaviour. Followed up with a 3 month phone call about progress and changes that may need to be made. Final follow up made after 6 months. **Duration** 6 months **Follow up from completion** 18 months **Theory** none stated
**Author** Holmes et al. 1996 [41] **Country** United States **Design** Quasi RCT	Total *n*=28; Intervention group: *n*=15 Control group: *n*=13 No details on gender and age	Physical Activity Diet	**Objective tools** • Fitness test (Techumseh Step Test for Cardiorespiratory Endurance) • Body composition (weight, height, BMI, • Blood pressure • Fasting bloods (lipids, glucose)	**Summary** Education provided in information handouts about healthy snacks to carry in their trucks, snack bags (with healthy foods), information sheets about healthier foods, exercise charts, list of tips for dining out, portion education, eating smart fat guide and a slide chart to help drivers understand food packaging information. **Duration** 26 weeks **Follow up from completion** Nil **Theory** none stated
**SINGLE ARM**				
**Author** Olson et al. 2009 [44] **Country** United States **Design** Pre-post	Total *n*=31, mean age 48.4 years (10.1) Male participants: 79%	Physical activity Diet Weight loss	**Objective tools** • Body composition (weight, height, BMI, • Blood pressure • Fasting bloods (lipids, glucose) • Fitness measures (strength, flexibility, 6 min walk test) **Self-Report** • Dietary behaviours (fruit and veg intake, high fat/high sugar foods) with “validated surveys”p.1236 • Physical activity recall interview	**Summary** Online training package to help truck drivers achieve and maintain a healthy body weight and work injury and crash free – done as a competition. Weight loss goals set for individuals at pre-intervention assessment (range 10-50lbs). Feedback sent to participants charting progress. Incentives included cash and jackets for winning teams. - Computer training units covered exercise, diet, and safety. Based on behavioural learning principles. Included stories from other drivers with personal health and safety experiences. Self-paced, estimated to take 3-4 hours to complete.63 sub-topics, with quizzes and testing included Self-monitoring activity/assignments included for support of transfer of training. Must complete with 80% correct and completed assignments. - Motivational interviewing – each driver had the opportunity for 4 motivational interview sessions with a health coach of 30-45min duration. - SHIFT program website. Included description of program, daily goals for diet, exercise, and safety, biweekly posts of competition status, and individual feedback, training tests, links to health and safety websites, description of scientific evidence. **Duration** 26 weeks (6 months) **Follow up from completion** Nil **Theory** Transtheoretical Model of Behaviour Change
**Author** Heaton et al. 2010 [45] **Country** United States **Design** Within subject cross-over pilot study	Total *n*=25, mean age 43 years (SD10.7), Male participants: “mostly men” % not reported	Sleep	**Objective tools** • Sleep actigraphy **Self-Report** • Karolinska Sleepiness Scale • Sleep quality (Likert scale) • Perceived control sleep (Likert Scale) • Attitude toward sleep (Likert scale)	**Summary** Participants were taught how to understand visual information from sleep actigraphy to improve their sleep behaviours. Drivers also completed sleep log diaries. Drivers either received the feedback first, then no feedback or no feedback then feedback. **Duration** 10 days each condition – 20 days total **Follow up from completion** N/A. **Theory** Theory of planned behaviour
**Study** Sorensen et. al. 2010 [46] **Country** United States **Design** Pre-post	Total *n*=277, mean age 48.5 (7.9) years Male participants: not reported	Diet Weight loss Smoking cessation	**Objective tools** • Blood pressure **Self-Report** • Self-reported height and weight to calculate BMI • Tobbaco use • Food choices (fruit and veg intake, sugary drinks)	**Summary** Participants received a tailored feedback report booklet targeted to freight drivers and targeted to the goal behaviour that the driver identified in baseline interview. Behaviours included smoking, fruit and veg consumption, sugary drinks and snacks, intention to lose weight. Participants received up to 5 phone call counselling sessions wherein the researcher would assist the driver to explore readiness for change and motivation, set and review goals identified in the feedback report. The driver would also be mailed an information brochure targeted to their health behaviour at each of these calls. Calls occurred over 4 month intervention period. **Duration** 4 week**s** **Follow up from completion** 6 months **Theory** Social cognitive theory and transtheoretical model
**Author** Thiese et al. 2015 [17] **Country** United States **Design** Pilot	Total *n*=12 Mean age 50.7 years (SD not provided) Male participants: 100%	Physical Activity Diet Weight loss	**Objective** • Body composition (weight, height, BMI) • Blood pressure • Fasting bloods (lipids, glucose) • Step count (pedometer) **Self-Report** • National Cancer Institute Automated Self-Administered 24 hour recall for food intake	**Summary** Given health education materials including audio, print, exercise equipment (resistance bands, dumbbells, yoga mat, pedometer) and healthy eating tools (snacking advice, on the go stove and pans, cook book). The participants worked with a health coach to develop three health related goals. Weekly check-ins in with health coach. **Duration** 12 weeks **Follow up from completion** Nil **Theory** Nil
**Author** Sendall et al. 2016 [47] **Country** Australia **Design** Single group pre-post and follow up	Total *n*=46 Male participants: 100%	Physical Activity Diet	**Self-Report** • Physical activity • BMI • Food diary	**Summar**y A co-design project between workplaces and the research team to create seven workplace health promotion interventions. These included posters, health options vendin machines, supply free fruit, 10000 step challenge, health eating and/or PA toolbox talks, healthy messages on payslips and a Facebook page **Duration** 26 weeks (6 months) **Follow up from completion** 12 weeks (3 months) and 24 weeks (6 months) **Theory** PAR process to engage stakeholders in the development, implementation and evaluation of collaborative solutions to a shared problem
**Author** Gilson et al. 2017 [16], 2016[31]* **Country** Australia **Design** Single group Pre-post and follow up	Total *n* = 19, mean age 44.4 years Male participants: 100%	Physical Activity Diet	**Objective tools** • Pedometer • Blood Pressure • Body Composition (BMI, waist circumference) **Self-Report** • Food diary	**Summary** An education program focussed on being physical active during rest break and improved diet (increased fruit and vegetable intake and reduce sugary foods/high fat/refined foods. Individualised to each driver on how to improve physical activity and dietary choices during intervention period. Participants also provided a resource pack with information on physical activity and diet, and provided a activity tracker and app. Participants were provided with financial incentives to remain engaged with the activity tracker and app. **Duration** 20 weeks **Follow up from completion** 8 weeks (2 months) **Theory** not based on any theory
**Author** Wilson et al. 2018 [48] **Country** United States **Design** Pre-post	Total *n* = 19, mean age 44.8 years (range 26-69 years), Male Participants: 100%	Weight loss	**Objective tools** • Body composition (weight, height, BMI) **Self-Report** • Modified Weight Efficacy Lifestyle Questionnaire	**Summary** Motivational interviewing was provided to participants for 2 hours/week for 4 weeks to help increase self-efficacy for weight loss and healthy eating. **Duration** 4 weeks **Follow up from completion** Nil **Theory** Social cognitive theory
**Author** Varela-Mato et al. 2018 [49] **Country** England **Design** Pre-post	Total *n*=72, mean age: 49.5 years (SD 5) Male participants: 100% male	Physical activity Diet Smoking cessation Alcohol consumption Sleep	**Objective tools** • Accelerometry • Body composition (weight, height, BMI) • Blood pressure • Fasting bloods (lipids, glucose) • Fitness measures (strength, flexibility, 6 min walk test) **Self-Report** • Anxiety and Depression – Hospital and Anxiety Depression Scale • Health Screen Questionnaire (past medical history, daily fruit and veg intake, smoking history, alcohol intake) • Sleep – daily log book	**Summary** Multi-component intervention consisting of: 1) 1:1 counselling on baseline results, discussed potential health changes (diet, PA) 2) SHIFT structured education program - small groups received 6-hour education (diet, PA, SB, lifestyle incl. alcohol, tobacco, sleep, stress) and their effect on health and work license. Designed to cover drivers' needs. 3) Cab workout - exercises for major muscle groups lasting 20min.. Advised to perform during breaks and long waits and unable to leave vehicle 4) Health coaching - Monthly catch up with health coach, discussing PA and diet. 5) Step challenge - based on Pedometers and 6) Healthy packed lunch scheme - drivers could pre-order a healthy lunch as alternative to service station food. **Duration** 12 weeks **Follow up from completion** 3 months **Theory** Grounded in health behaviour change theories including Bandurra social cognitive model and Leventhal common sense model
**Author** Olson et al. 2020 [50] **Country** United States **Design** With-in subject repeated design	Total *n*=16 Mean age 48 years (SD10.26) Intervention *n*=8 Control *n*=8 Male participants: 81%	Sleep Physical Activity Diet	**Objective tools** • Accelerometry • Body composition (weight, height, BMI, • Blood pressure **Self-Report** • Pittsburgh Sleep Quality Index • Healthy Physical Activity Scale (includes dietary measures)	**Summary** Participants exposed to three different sleeping conditions (A, B, C for 2-3 weeks per condition) and completed a behavioural sleep-health program which focussed on sleep hygiene, physical activity and dietary choices. The behavioural sleep-health program was based on the SHIFT program (Reference Olson 2009). **Duration** Condition A: 2-3 weeks Condition B: 2-3 weeks Condition C: 3 months **Follow up from completion** Nil **Theory** Not reported

^$^Puhkala 2015 and 2016 combined as one study

*Gipson 2016 and 2017 combined as one study

### Population

The 19 studies had a total sample of *n*=2,137 participants. Among the k=14 studies that reported gender, 94.3% of participants were male, and the mean age was 46 years.

### Interventions

A detailed summary of the individual study and intervention characteristics is provided in Table 1: Overview of Included Studies. Interventions ranged in duration from two weeks [18] to 12 months [32, 33]. Most studies (k=14) delivered multi-component interventions that targeted numerous health behaviours including increasing PA (k=11) [16, 18, 31–34, 37, 40, 42–44, 47, 49], increasing fruit and vegetable intake (k=11) [16–18, 31–34, 40–42, 44, 46, 47], losing weight (k=2) [44, 46], improving sleep behaviours (k=5) [38, 42, 44, 45, 49] and ceasing smoking (k=3) [40, 46, 49]. Individualised health education sessions were provided in 11 studies [18, 32, 33, 37–42, 44, 46, 50] and group education sessions by three studies [16, 31, 34]. This was supported by educational resources, including physical materials and handouts, exercise equipment [16, 17, 31, 40, 41, 45, 46, 49], online training packages/web applications [18, 34, 39, 42–44, 47, 50], and audio materials [17]. Motivational interviewing (MI) was provided by nearly 50% of the included studies (k=8). The volume of MI varied between studies, providing MI in a single instance to facilitate initial goal-setting (k=2) [40, 46], whilst others (k=6) provided MI throughout the intervention. Eleven studies described a theoretical framework for the intervention. Theories used included Social Cognitive Theory [34, 46, 48, 49], Behavioural Self-Monitoring Theory [44], the Health Action Process Approach [32, 33], the Transtheoretical Model [38, 46], the Social Contextual model [37], the Health Belief Model [39] and Self-Efficacy theory [37].

### Outcomes

Studies reported on a range of outcomes, broadly categorised as either a) health behaviours or b) cardiometabolic health biomarkers.

### Health behaviour outcomes

Physical activity was assessed in k=13 studies [16–18, 31–34, 37, 40, 42, 44, 47, 50]. Assessment methods included pedometers (k=3) [16, 17, 31–33], accelerometers (k=4) [34, 42, 49, 50], and self-report measures (k=8) [17, 18, 32, 33, 37, 40, 44, 47, 50]. Fitness testing was conducted in k=5 [40, 41, 43, 44, 49] studies. Dietary outcomes were assessed in k=13 studies [16–18, 31–34, 39, 40, 42, 44, 46, 48–50] using either validated published questionnaires (k=6) [17, 18, 39, 44, 48, 50] or study developed self-report tools (k=7) [16, 31, 40–42, 46, 47, 49]. Sleep outcomes were assessed in k=6 studies [18, 32, 38, 42, 45, 50] using actigraphy (k=3) [38, 42, 45], validated sleep scales (k=5) [18, 38, 42, 49, 50] or self-reported sleep quality (k=4) [38, 42, 45, 49]. Smoking frequency was assessed in k=3 [40, 46, 49] and alcohol use in k=1 [49].

### Cardiometabolic health biomarkers

BMI was assessed in k=15 studies [16–18, 31–34, 39–42, 44, 46–50]; blood pressure in k=10 [16–18, 31–33, 40, 41, 44, 46, 49, 50]; cholesterol in k=8 studies [17, 18, 32, 33, 40, 41, 44, 49, 50], fasting blood glucose by k=8 studies [17, 18, 32, 33, 40, 41, 44, 47, 49]; and anthropometric (e.g., body fat %) by k=2 studies [18, 32, 33].

There were sufficient data to perform meta-analyses of intervention versus comparison groups for the following outcomes: PA, weight, total cholesterol, HDL cholesterol, systolic blood pressure, diastolic blood pressure and fasting blood glucose. For meta-analyses of change between pre-and post-intervention, there were sufficient data for the following outcomes: PA, fruit and vegetable intake, weight, BMI, sleep quality, sleep duration, total cholesterol, HDL cholesterol, LDL cholesterol, systolic blood pressure, diastolic blood pressure, and fasting blood glucose.

### Risk of bias in studies

The CASP for RCTs [21] was used to assess risk of bias. The results of the risk of bias assessments are shown in Table 2. Nearly all the RCTs (including cluster RCTs) were considered moderate quality [18, 32–34, 38, 42, 43] scoring ≥ 6/11on the CASP tool. There were issues relating to blinding of participants, risk assessment and local application. There was greater variability in the quality of the Quasi-RCTs (k=4); with scores ranging from 1/11 (scoring only for focussed research question) [44] to 4/11 [46] with issues relating to randomization, attrition, blinding, baseline differences, reporting of results, risk assessment and local application.

**Table 2:**
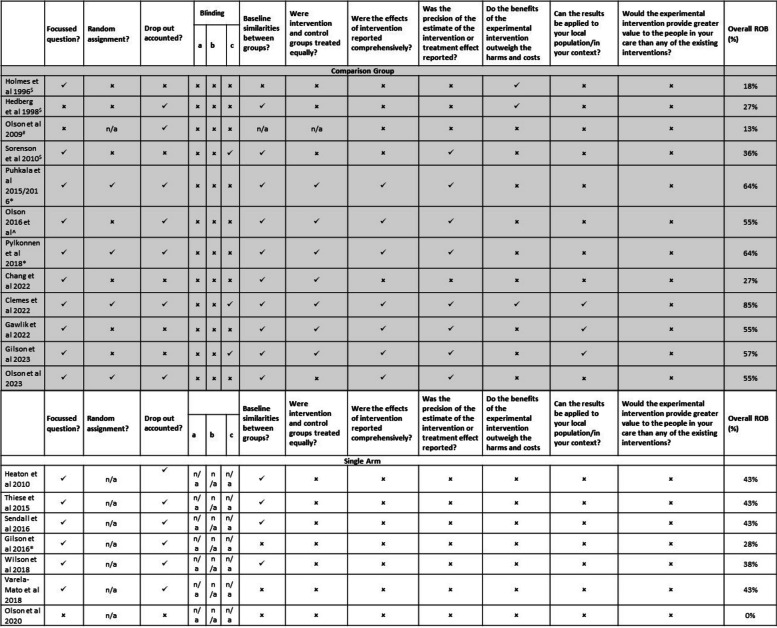
Risk of bias of included studies

*=RCT, ^=Cluster-RCT, $=Quasi-RCT, #=pre/post, n/a = not applicable, ü = yes, û = no/can’t tell

Of the single-arm studies, 7 of the 11 CASP questions were applicable (criteria relating to group assignment, baseline differences between groups and level of care received were all not applicable.) Scores ranged from 0/7 [50] to 3/7 [17, 45, 47, 49]. Typically, single-arm studies performed poorly on reporting results, an estimate of the effect of the intervention, risk assessment and local application.

### Results of syntheses

We grouped studies according to their design. Results from intervention vs control meta-analyses are presented first, followed by results from pre-post-intervention meta-analyses. Finally, narrative syntheses are presented for the outcomes that could not be meta-analysed (Summary of Findings Table – Table 3).

**Table 3:** Summary of findings table

Outcomes	**Anticipated absolute effects**^**k**^ (95% CI)		Relative effect (95% CI)	№ of participants (studies)	Certainty of the evidence (GRADE)	Comments
	**Risk with usual practice**	**Risk with health behaviour interventions**				
Physical Activity	-	SMD **0.18 SD higher** (0.05 higher to 0.41 higher)	-	1085 (5 RCTs)	⨁◯◯◯ Very low^a,b,c,d^	Health behaviour interventions may increase physical activity in truck drivers but the evidence is very uncertain
Weight	-	SMD **0.15 SD higher** (0.07 lower to 0.38 higher)	-	1043 (4 RCTs)	⨁◯◯◯ Very low^a,b,c,d^	The evidence is very uncertain about the effect of health behaviour interventions on weight in truck drivers
Total cholesterol	-	SMD **0.01 SD higher** (0.3 lower to 0.31 higher)	-	930 (3 RCTs)	⨁◯◯◯ Very low^b,c,e,f^	Health behaviour interventions may have little to no effect on total cholesterol but the evidence is very uncertain
HDL cholesterol	-	SMD **0.02 SD lower** (0.16 lower to 0.12 higher)	-	1043 (4 RCTs)	⨁⨁◯◯ Low^a,c^	The evidence suggests that health behaviour interventions results in little to no difference in HDL cholesterol
Systolic Blood Pressure	-	SMD **0.11 SD higher** (0.45 lower to 0.66 higher)	-	480 (2 RCTs)	⨁◯◯◯ Very low^b,c,e,f^	Health behaviour interventions have little to no effect on Systolic Blood Pressure but the evidence is very uncertain
Diastolic Blood Pressure	-	SMD **0.36 SD higher** (0.63 lower to 1.34 higher)	-	480 (2 RCTs)	⨁◯◯◯ Very low^b,c,e,g^	The evidence is very uncertain about the effect of health behaviour interventions on Diastolic Blood Pressure
Fasting Blood Glucose	-	SMD **0.21 SD higher** (0.14 lower to 0.57 higher)	-	593 (3 RCTs)	⨁◯◯◯ Very low^a,c,d,h^	The evidence is very uncertain about the effect of health behaviour interventions on Fasting Blood Glucose
Smoking				446 (4 RCTs)	⨁◯◯◯ Very low^c,d,i,j^	*n*=3 reported no significant changes following intervention whilst *n* = 1 study reported associations between process outcomes (e.g., engagement with program literature, number and perception of program phone calls, etc.) and the likelihood of quitting but did not report aggregated group data on the overall change in smoking behaviours
Alcohol				72 (1 Pre-Post study)	⨁◯◯◯ Very low^c,d,i,j^	No statistically significant difference in units of alcohol consumed per week (*p*=0.130) following a multi-component PA and diet-focused intervention

GRADE Working Group grades of evidence

High certainty: we are very confident that the true effect lies close to that of the estimate of the effect

Moderate certainty: we are moderately confident in the effect estimate: the true effect is likely to be close to the estimate of the effect, but there is a possibility that it is substantially different

Low certainty: our confidence in the effect estimate is limited: the true effect may be substantially different from the estimate of the effect

Very low certainty: we have very little confidence in the effect estimate: the true effect is likely to be substantially different from the estimate of effect

*CI* Confidence interval, *SMD* Standardised mean difference

^a^Downgraded one on risk of bias because of issues with blinding

^b^downgraded two due to considerable heterogeneity

^c^downgraded one due to indirectness in application to settings

^d^Wide confidence interval

^e^downgraded two as both studies at high risk of bias

^f^downgraded two due to very wide confidence interval

^g^downgraded three due to extremely wide confidence interval

^h^downgraded one due to substantial heterogeneity

^i^downgraded due to high risk of bias

^j^unable to assess inconsistency

^k^The risk in the intervention group (and its 95% confidence interval) is based on the assumed risk in the comparison group and the relative effect of the intervention (and its 95% CI)

### Meta-analyses – intervention vs comparison groups

**Fig. 2:**
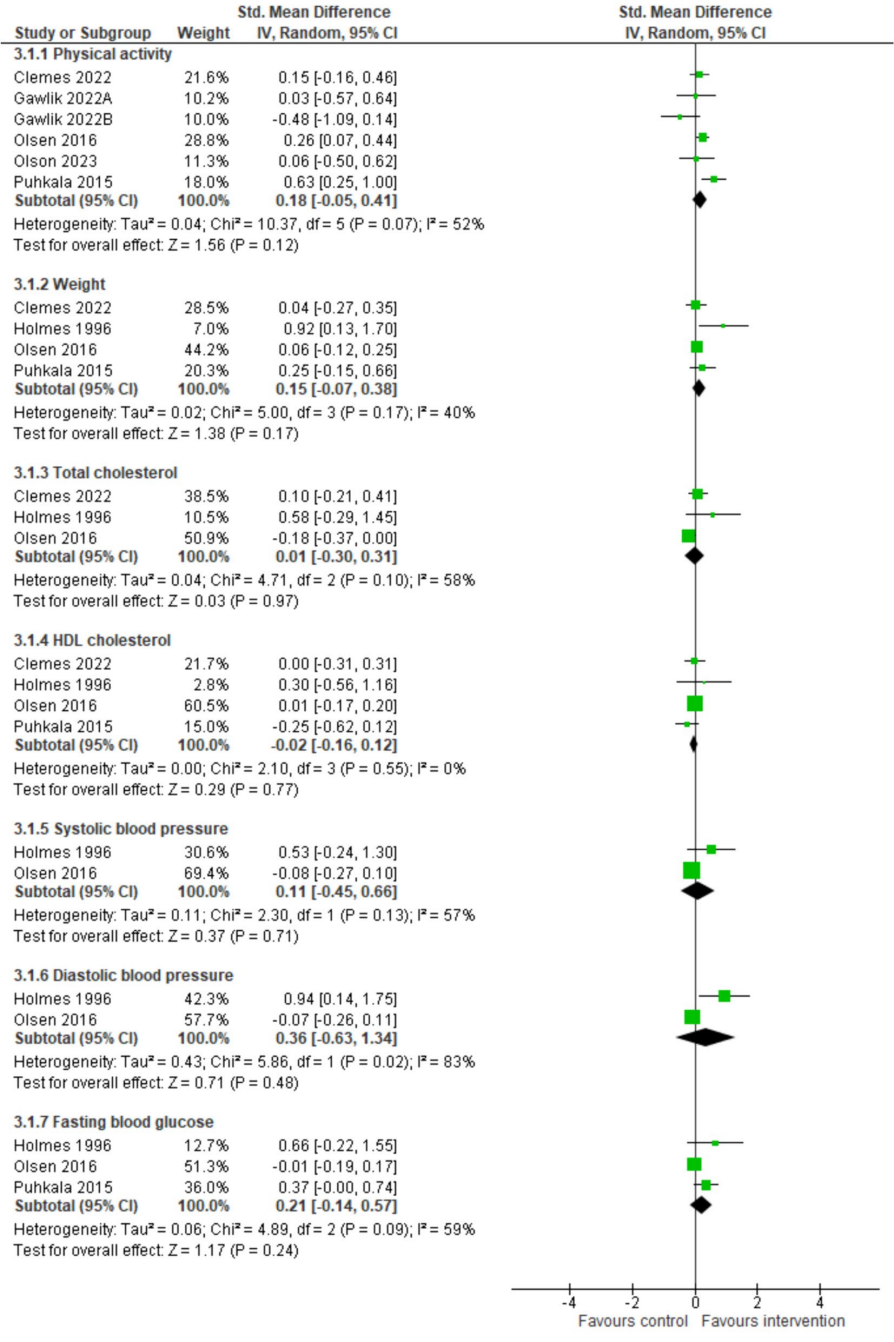
Meta-analysis results of intervention versus comparison conditions*

Figure 2 summarises the findings of meta-analyses comparing intervention to comparison groups. There was no significant effect in favour of the interventions for PA, (k=5, *n*=1085, SMD=0.18 [95% CI=-0.05, 0.41], *I*^*2*^=52%, *p*=0.12, very-low certainty evidence). No effect was found for total weight reduction (k=5, *n*=1043, SMD=0.15, [95% CI=-0.07, 0.38], *I*^*2*^=40%, *p*=0.17), total cholesterol (k=3, *n*=930, SMD=0.01, [95% CI=-0.30, 0.31], *I*^*2*^=58%, *p*=0.97), HDL (k=4, *n*=1043, SMD=-0.02, [95% CI=-0.16, 0.12], *I*^*2*^=0%, *p*=0.77), systolic blood pressure (k=2, *n*=479, SMD=0.11, [95% CI=-0.45, 0.66], *I*^*2*^=57%, *p*=0.71)*,* diastolic blood pressure (k=2, *n*=479, SMD=0.36, [95% CI=-0.63, 1.34], *I*^*2*^=83%, *p*=0.48)*,* and fasting blood glucose (k=3, *n*=586, SMD=0.21, [95% CI=-0.14, 0.57], *I*^*2*^=59% *p*=0.24). There was insufficient data for meta-analyses of dietary intake, sleep, smoking, and alcohol use*.* Certainty of evidence was low for HDL, and very-low for all other outcomes.

### Meta-analysis of change between pre-and post-intervention

**Fig. 3:**
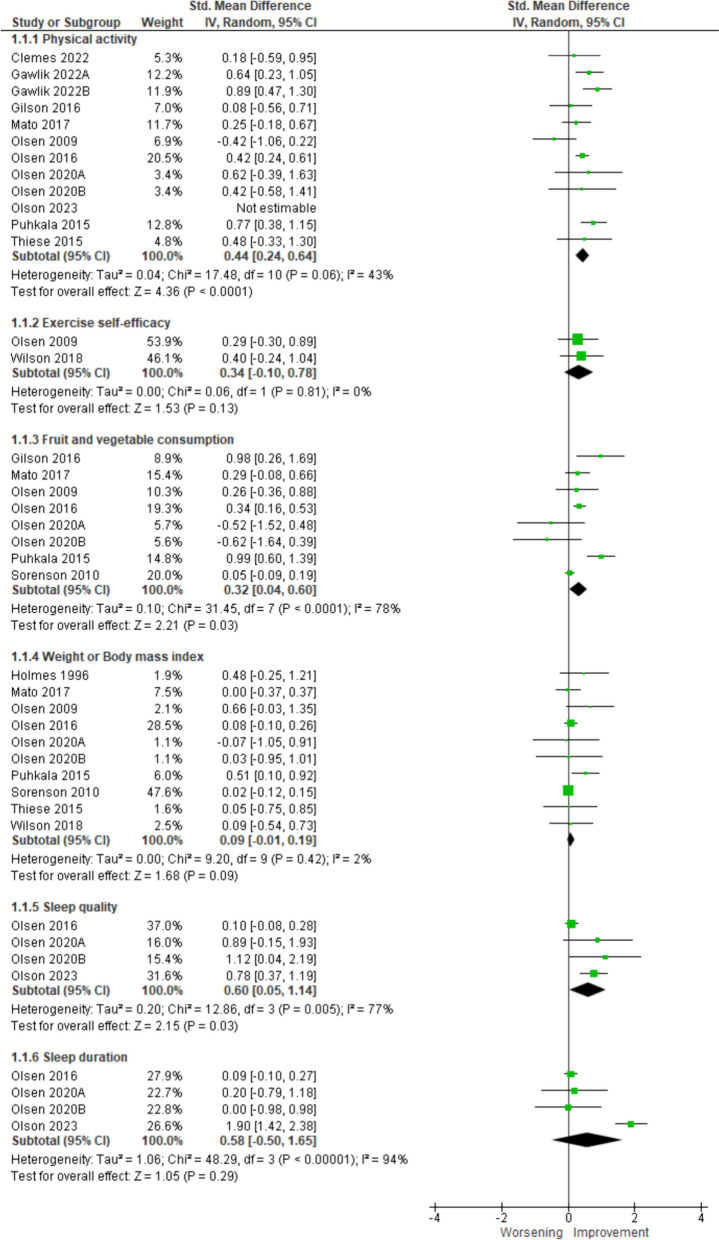
Meta-analyses of change in outcomes between pre-and post-intervention for PA, fruit and vegetable consumption, weight, BMI, sleep quality and sleep duration

Pre-post-intervention group changes were calculated for the following health behaviours: PA, dietary intake, sleep, and weight and cardiometabolic biomarkers of health, including total cholesterol and fasting blood glucose. These are represented graphically in Fig. 3. There was a moderate increase in PA from pre- to post-intervention (k=12, *n*=504, SMD=0.44 [95% CI=0.24,0.64], *I*^*2*^=43%, *p*<0.0001). Results of subgroup analyses showed no difference between self-reported (SMD=0.52 [95%CI=0.28, 0.76]) and accelerometer-assessed PA (0.19 [95%CI=-0.19, 0.51]; test for subgroup differences: χ^2^ =2.58, *p*=0.11, I^2^=61% – Supplementary File 5.

A small, significant improvement was observed for fruit and vegetable consumption from pre- to post-intervention (k=8; *n*=1598; SMD 0.32, *p*=0.03). There was no significant change in sleep quality, sleep duration, weight, BMI, total cholesterol, HDL, LDL, systolic blood pressure, diastolic blood pressure, and fasting blood glucose – Supplementary File 6.

### Narrative summary for outcomes that could not be meta-analysed

#### Smoking

The four studies that measured smoking as an outcome (k=3) reported no significant changes following intervention [40, 41, 49]. One study [46] reported associations between process outcomes (e.g., engagement with program literature, number and perception of program phone calls, etc.) and the likelihood of quitting but did not report aggregated group data on the overall change in smoking. In the study by Sorenson et al. [46], at post-intervention the smoking cessation and weight management group were significantly more likely to have quit smoking compared to initial smokers in the control condition (quit rate of 23.9% vs 9.1%, respectively).

#### Alcohol use

Only one study [49] reported on alcohol use. In a pre-post design, Varela-Mato et al. [49] observed no statistically significant difference in units of alcohol consumed per week (*p*=0.130) following a multi-component PA and diet-focused intervention.

## Discussion

This systematic review and meta-analysis assessed the effectiveness of interventions for health behaviours and cardiometabolic biomarkers of health in truck drivers. Only 19 studies were identified, with most of this research conducted on middle-aged male truck drivers from the US. Various intervention strategies were used, including goal-setting, lifestyle counselling, motivational interviewing, health-behaviour education and training. Meta-analyses demonstrated a small effect on fruit/vegetable consumption for within-group trials. Effects were in a favourable direction for physical activity, other behavioural and cardio-metabolic outcomes but were not statistically significant. The studies included in this review generally had a high risk of bias, resulting in an overall poor quality of evidence. Additionally, the certainty of evidence for each outcome was determined to be low to very low.

The systematic review findings suggest that health intervention programs for truck drivers may be effective in promoting physical activity and fruit and vegetable consumption. However, this result needs to be interpreted with substantial caution. First, positive effects of interventions for these outcomes could only be established in pre-post analyses, considering within sample change only. In contrast, there was no evidence for effectiveness of health behavioural interventions on physical activity, fruit and vegetable consumption (or any other outcome), compared to control conditions. As such, pre-post differences in physical activity or dietary behaviour may be driven in part by confounding factors such as the Hawthorne effect, rather than reflecting efficacy of the intervention per se. In addition, our confidence in these findings is impaired by the very-low certainty of evidence for each of the effects. There is a critical need for further research to develop our understanding of the effectiveness of interventions for these and other health outcomes in truck drivers.

Likewise, the meta-analysis findings showed that interventions appear to be ineffective in reducing weight or improving cardiometabolic health biomarkers. Despite these results being from between-group trials, the low certainty of evidence for these findings makes interpretation challenging, but it is possible that the short duration of interventions and follow-up periods may not be sufficient to lead to changes in downstream health outcomes such as weight, BMI, or cardiometabolic biomarkers [51]. A recent systematic review of systematic reviews of wearable activity trackers similarly reported strong evidence of improvements in physical activity behaviour but not in cardiometabolic health biomarkers [52].

It is important to acknowledge that the limited number of studies identified in this review (k=19) may have contributed to our findings of no significant difference for many outcomes examined. In general, the meta-analysis effects for behavioural and cardiometabolic markers were in a favourable direction, but statistically non-significant. In a number of cases, the effect sizes were large enough to be meaningful, should they be true effects (e.g. SMD >0.2 for diastolic blood pressure, fasting glucose and cholesterol, and SMD >0.5 for sleep quality and sleep duration). However, given the very-low to low certainty of evidence, we may expect effect estimates and confidence intervals to change with further research.

For an industry as vital as the transport industry, the body of evidence for health interventions for truck drivers is surprisingly small compared to other occupations. For example, a 2019 systematic review of health interventions for nurses identified 136 studies, including 52 RCTs [53]. Moreover, a recent umbrella review of health interventions for office workers identified 23 previous systematic reviews [54], with 517 component studies (unadjusted for overlap). The lack of research on truck drivers compared to other occupational groups may be due to the unique challenges of the profession, such as working long hours in varying locations, differing demands, and working conditions [55]. This industry's difficulty in engaging with health interventions may reflect the lack of sufficient funding for health promotion in this at-risk occupation. The transport industry, however, is beginning to take a more active interest in promoting the health and well-being of drivers [56]. The “Driving Health” study is an example of research and industry collaborating to develop evidence-based wellbeing strategies to benefit Australian drivers [56]. This project conducted research to determine the major areas of health concern for drivers, and to co-develop health programs that could be implemented into workplaces [56]. So far the project has released a free online training resource for supervisors and managers to help better understand the factors that influence driver health and wellbeing [56].

A key strength of our study is that it is the first meta-analysis of the effects of health behaviour interventions for truck drivers. We included a wide range of target health behaviours, comprehensively synthesising the current evidence base. In addition, we adhered to rigorous systematic review and meta-analysis approaches, including meta-analysis, risk of bias assessment and assessing the certainty of the evidence. A limitation was that we may have missed studies published in non-English languages (though there were none, to our knowledge), or grey literature sources such as industry reports. The most important limitations of our study related to the small evidence base we had to work with. For example, there were insufficient studies to conduct subgroup analyses based on intervention characteristics, which would have been valuable to inform future intervention development.

The health risks associated with the truck driver occupation are significant and varied, ranging from physical inactivity to poor diet and shift work. Further to this, there are wider safety implications associated with truck driver health, with evidence demonstrating drivers with obesity have higher rates of accidents [57]. Given the importance of the industry, future research must prioritise the development of practical and scalable healthy lifestyle interventions to promote the health and well-being of truck drivers. The majority of truck drivers are men, who are widely recognised as being difficult to engage in preventive health programs [55]. Most studies identified in this review have taken a personal responsibility approach, i.e. the interventions were framed in terms of teaching and encouraging individuals to take responsibility for their own health outcomes through their choices and behaviours [58]. Further to this, recent statistics suggest that more females are working in truck driver roles with growth in the US from 8% in 2018 to 14% in 2022 [59] and from 3.3% in 2016 to 4.3% in 2021 in Australia [60]. However, such interventions may not account for the broader workplace and environmental factors that contribute to poor health outcomes among truck drivers.

Future interventions should adopt a multi-level approach that considers the biological, social, and environmental determinants of health. This could involve workplace policies that encourage healthy behaviours, such as providing subsidised lunches with fruit and vegetables to ensure drivers have access to nutritious food even on the road. Companies could also facilitate access to exercise facilities at major truck stops to support for physical activity during rest breaks. An important component to planning future interventions would be to work with end-users and stakeholders using a co-design process. This approach helps tailor the interventions to the drivers’ needs and increases the likelihood of successful implementation and ongoing sustainability [61]. There also needs to be consideration for how these interventions might work in low-middle income countries (LMIC). All the studies included in this review were from very-high income countries (VHIC). Truck drivers from LMIC have similar health risks and disease burden as those from VHIC. However despite these similarities, there is a significant health investment and assistance gap for drivers from LMIC locations [62]. By addressing these broader factors, interventions may be more successful in promoting the health and well-being of truck drivers, including those who may be traditionally harder to engage in lifestyle programs. Furthermore, this approach could lead to the development of more sustainable and effective interventions that can benefit the wider truck driver population.

## Conclusion

This systematic review highlights the limited evidence available on the effectiveness of health interventions for truck drivers. Results suggest that interventions may provide small-moderate beneficial effects on physical activity, as well as fruit and vegetable consumption, over time (i.e., within-sample) but not relative to control conditions. Results from both within- and between-group meta-analyses did not support the effectiveness for weight loss nor cardiometabolic health biomarkers. The very-low to low certainty of evidence for each of these effects means that further research is very likely to have an important impact on our confidence in effect estimates, and perhaps the estimates themselves. This review also underscores the need for further research that takes a multi-level approach to promote the health and well-being of truck drivers, considering the workplace and environmental factors that may contribute to poor health outcomes. As the transport industry increasingly recognises the importance of addressing driver health, future research should aim to develop and implement sustainable and effective interventions that address the unique challenges facing truck drivers. Doing so could have significant benefits, not just for the health and well-being of individual drivers, but for the transport industry as a whole.

## Supplementary Information

Supplementary Material 1.

Supplementary Material 2.

Supplementary Material 3.

Supplementary Material 4.

Supplementary Material 5.

Supplementary Material 6.

## Data Availability

Availability of the data extraction is available in supplementary file 3. Availability of the meta-analyses calculations in RevMan5 are available upon reasonable request.

## Abbreviations

BMI: Body Mass Index
PA: Physical Activity
CI: Confidence interval
RCT: Randomised controlled trial
MD: Mean differences
SMD: Standardised mean differences
