# Supplementary figures and images for: Keep on truckin’: how effective are health behaviour interventions on truck drivers’ health? A systematic review and meta-analysis
Source: BMC Public Health. 2024 Sep 27;24:2623. doi: 10.1186/s12889-024-19929-1 (PMC11438120; doi:10.1186/s12889-024-19929-1)

Supplementary File 1: Database searches

**Medline Search**


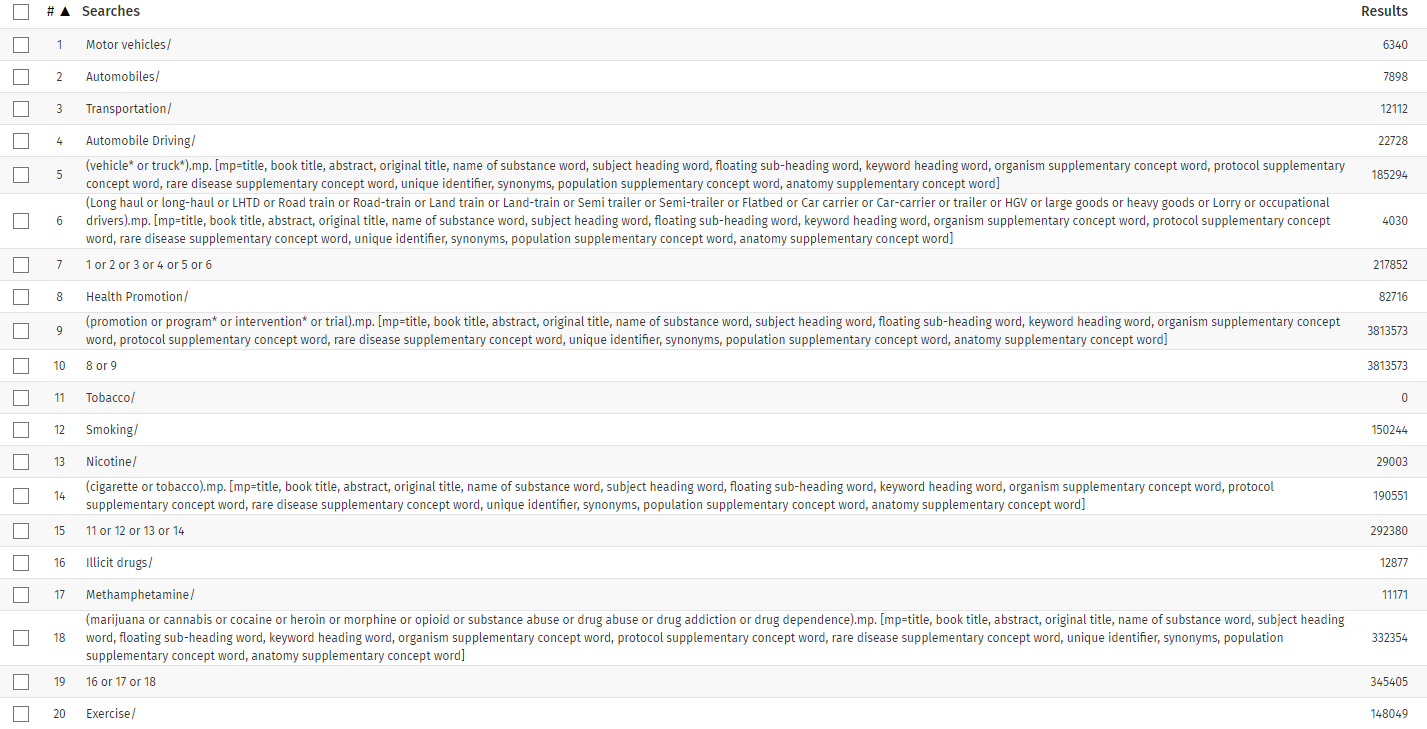


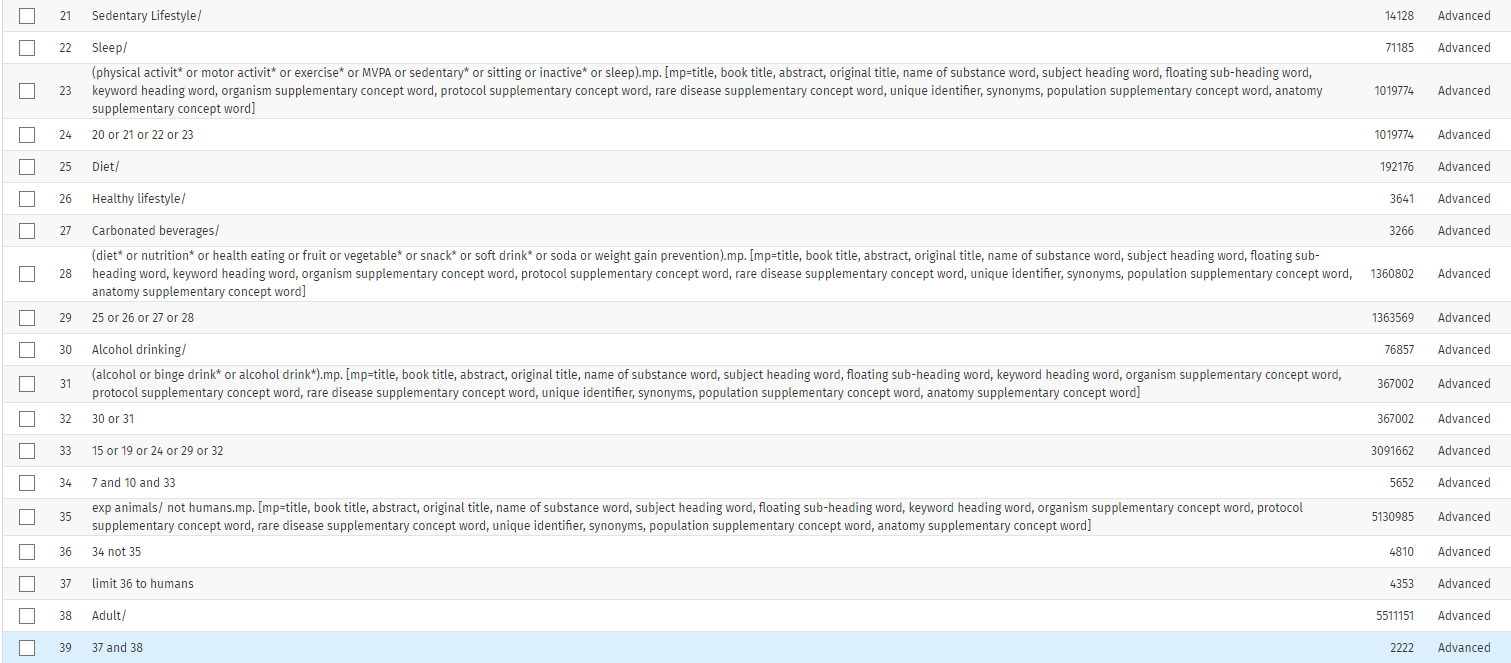


Embase search


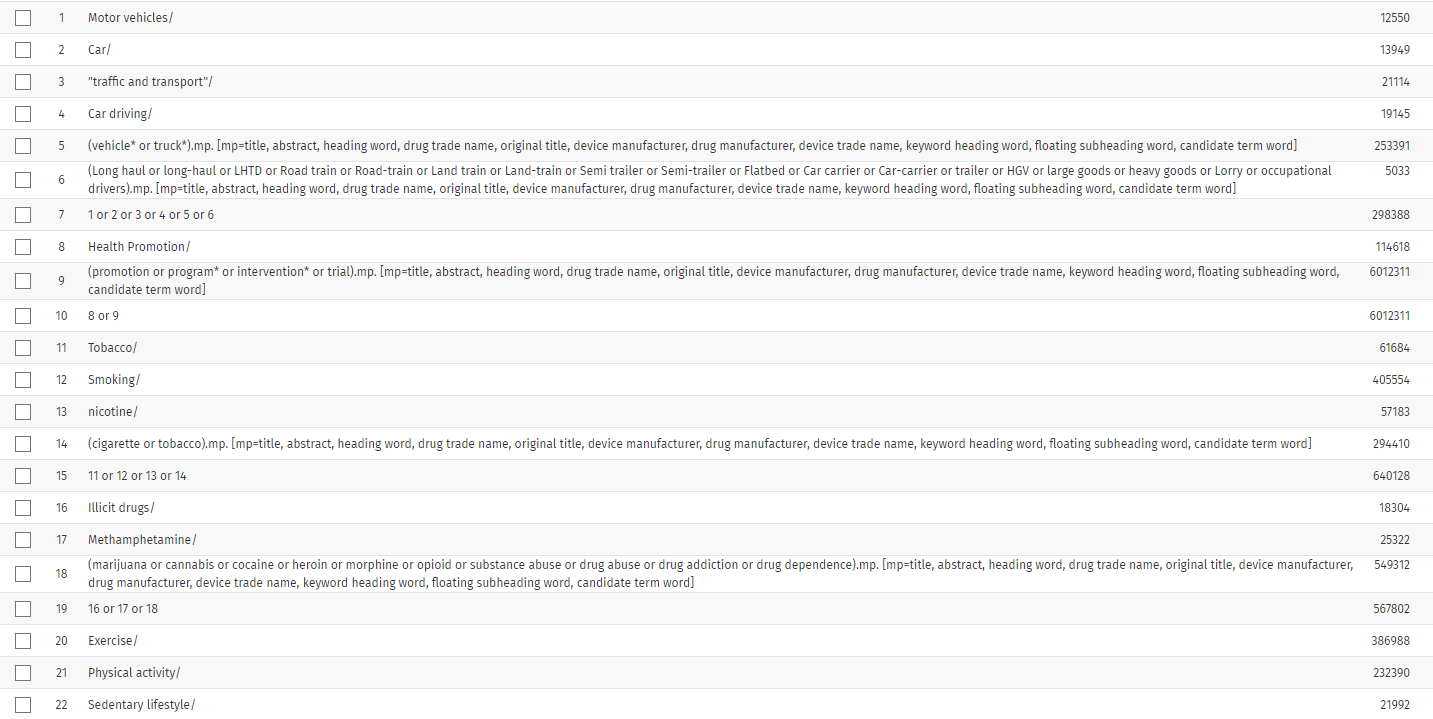


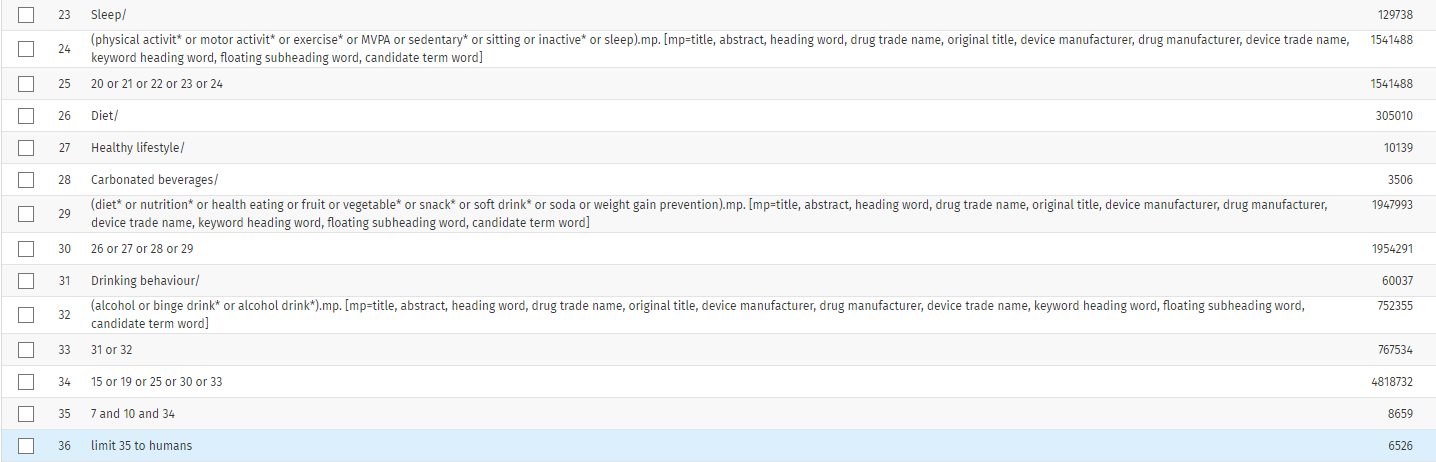


Emcare search


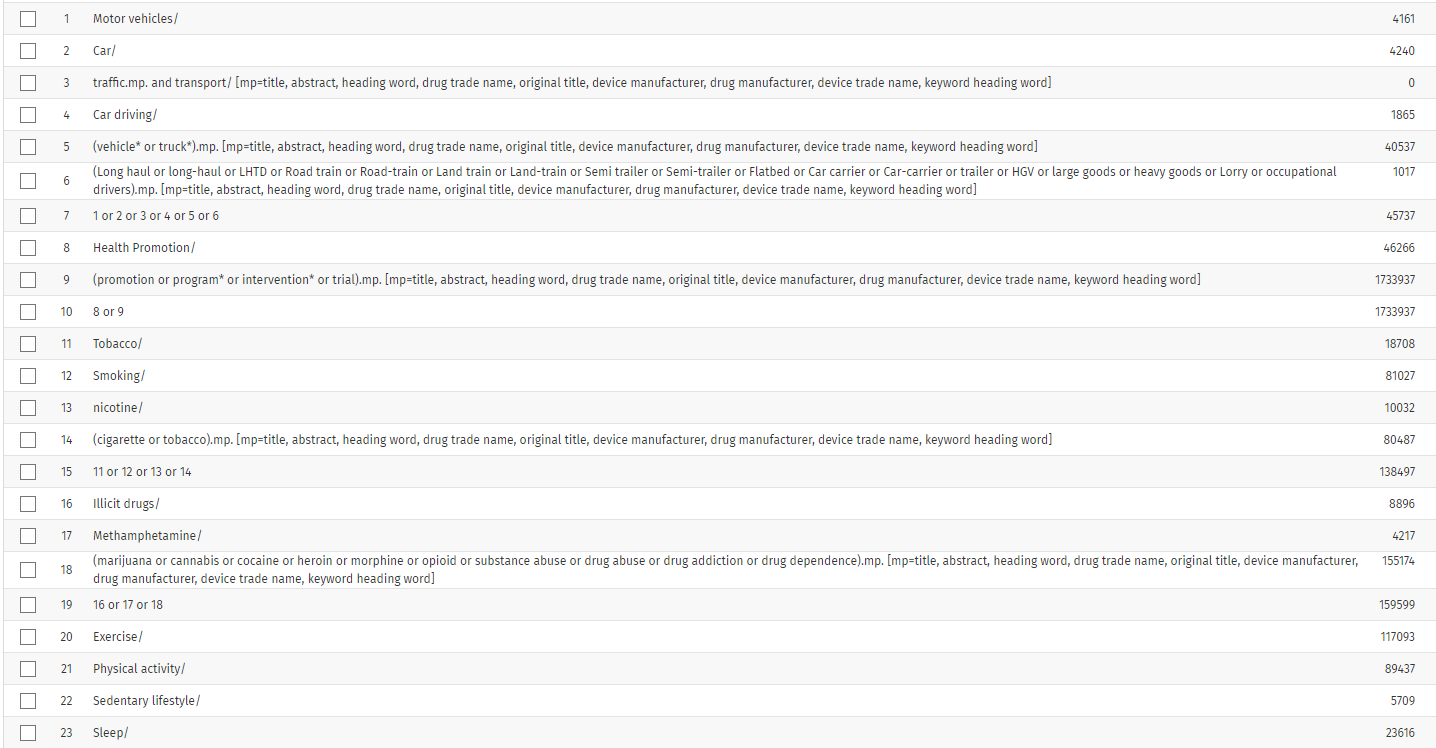


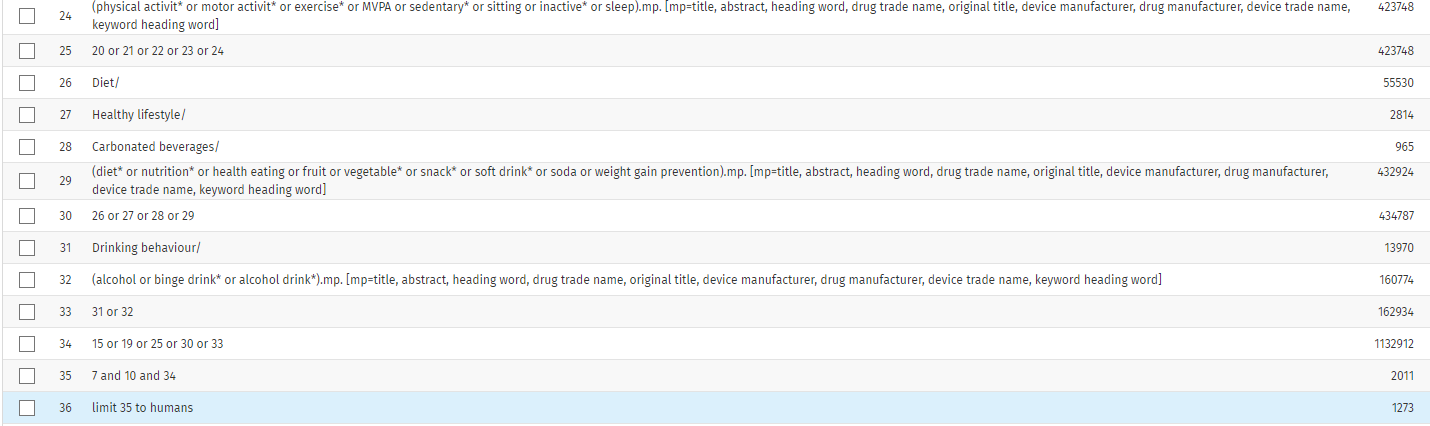

Supplement: Supplementary file 1 — Supplementary Material 1. [file 12889_2024_19929_MOESM1_ESM.docx]

Supplementary File 5: Subgroup analyses of physical activity assessment type


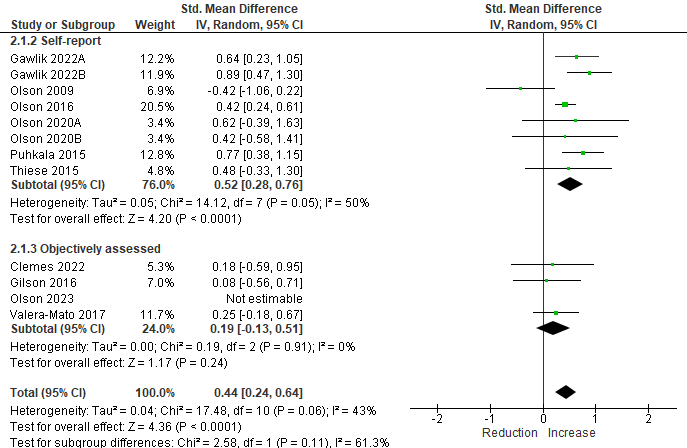

Supplement: Supplementary file 5 — Supplementary Material 5. [file 12889_2024_19929_MOESM5_ESM.docx]
