# Supplementary material for: Keep on truckin’: how effective are health behaviour interventions on truck drivers’ health? A systematic review and meta-analysis
Source: BMC Public Health. 2024 Sep 27;24:2623. doi: 10.1186/s12889-024-19929-1 (PMC11438120; doi:10.1186/s12889-024-19929-1)
Supplement: Supplementary file 2 — Supplementary Material 2. [file 12889_2024_19929_MOESM2_ESM.docx]

Supplementary File 2: Inclusion/Exclusion Checklist for screening

| **Inclusion Criteria** | **Exclusion Criteria** |
| --- | --- |
| Health based intervention | Observational studies of truck driver health outcomes |
| Data relating to truck drivers/freight drivers | Not full-text |
| Studies published in English |  |
| Adult truck drivers |  |
| Any comparator acceptable |  |
