# Supplementary material for: Keep on truckin’: how effective are health behaviour interventions on truck drivers’ health? A systematic review and meta-analysis
Source: BMC Public Health. 2024 Sep 27;24:2623. doi: 10.1186/s12889-024-19929-1 (PMC11438120; doi:10.1186/s12889-024-19929-1)
Supplement: Supplementary file 3 — Supplementary Material 3. [file 12889_2024_19929_MOESM3_ESM.docx]

Supplementary File 3: Data Extraction Tool

| **Label** | **Data** | **Reference e.g. Virgara et al 2020, “The effectiveness of health education” BMC** |
| --- | --- | --- |
| publication status | 1. published  2. in press/accepted for publication |  |
| design of study | 1. Randomised controlled trial  2. Cluster RCT  3. Quasi RCT  4. pre-post measures  5. pilot study  6. within subject 2 conditions |  |
| participant selection | 1. convenience sample of non-random participants  2. random selection  3. representative sample  4. other |  |
| behaviours targeted in intervention | 1. PA  2. diet  3. weight loss  4. smoking cessation  5. alcohol consumption  6. sleep |  |
| sample size at commencement of study |  |  |
| average age of sample |  |  |
| % male participants |  |  |
| % population white |  |  |
| country of origin | 1. USA 2. Australia 3. England 4. Singapore 5. NZ 6. Canada 7. Belgium 8. Norway 9. Taiwan 10. South Korea 11. Netherlands 12. Other |  |
| Study Setting | Write descriptively |  |
| Intervention Setting | Write descriptively |  |
| type of measures used | 1. self-report 2. objective |  |
| Self-report physical activity tool used | 1. IPAQ (short form) 2. IPAQ (long form) 3. BRFSS 4. 2D PAR 5. NHANES III 6. Active Australia 7. Godin 8. 7-day recall 9. Not required 10. Other - record which tool |  |
| Reliability of self-report PA tool reported | 1. yes 2. no 3. not reported |  |
| validity of self-report PA measure reported | 1. yes 2. no 3. not reported |  |
| objective physical activity measure | 1. accelerometer 2. pedometer 3. direct observation 4. other - record |  |
| Physical activity mode assessed | 1. overall physical activity 2. walking 3. leisure time physical activity 4. vigorous intensity physical activity 5. moderate intensity physical activity 6. light intensity physical activity 7. Other - record details |  |
| reliability and/or validity of Objective Measured PA | 1. yes 2. no 3. not reported |  |
| other outcome measured 1 (write descriptively) |  |  |
| name of tool used 1 (write descriptively) |  |  |
| reliability and/or validity of measurement tool reported 1 | 1. yes 2. no 3. not reported |  |
| other outcome measured 2 (write descriptively) |  |  |
| name of tool used 2 (write descriptively) |  |  |
| reliability and/or validity of measurement tool reported 2 | 1. yes 2. no 3. not reported |  |
| other outcome measured 3 (write descriptively) |  |  |
| name of tool used 3 (write descriptively) |  |  |
| reliability and/or validity of measurement tool reported 3 | 1. yes 2. no 3. not reported |  |
| Other measures | Other outcome measure, name of tool, reported reliability and/or validity |  |
| Number in Intervention group |  |  |
| Number in control group |  |  |
| Type of baseline measure | write descriptively |  |
| Type of introduction session to intervention | write descriptively |  |
| duration of intervention (weeks) |  |  |
| number of follow up time points (other than immediately post intervention) |  |  |
| length of time from post-test measure to first follow up (weeks) |  |  |
| length of time from post-test measure to second follow up (weeks) |  |  |
| length of time from post-test measure to third follow up (weeks) |  |  |
| intervention tailored to individual | 1. Yes 2. No |  |
| intervention developed based on theory? | 1. Yes 2. No |  |
| If yes (1), Which theory | 1. SCT 2. TPB 3. TTM (incl SOC) 4. SOC (not include TTM) 5. Self-management theory 6. Social ecological model 7. Protection motivation theory 8. Goal-setting theory  9. Health beliefs model 10. Other (write out) |  |
| Sample size at end of study (intervention group) |  |  |
| Sample size at end of study (control group) |  |  |
| Physical Activity Outcome (name outcome) | 1. Yes - significant improvement compared with control (↑)  2. No significant difference between intervention and control (-) 3. No - control group improved (↓)  Provide Scores e.g. MD, p value etc |  |
| Diet outcomes | 1. Yes - significant improvement compared with control (↑)  2. No significant difference between intervention and control (-) 3. No - control group improved (↓)  Provide Scores e.g. MD, p value etc |  |
| Weight loss outcomes | 1. Yes - significant improvement compared with control (↑)  2. No significant difference between intervention and control (-) 3. No - control group improved (↓)  Provide Scores e.g. MD, p value etc |  |
| Smoking cessation outcomes | 1. Yes - significant improvement compared with control (↑)  2. No significant difference between intervention and control (-) 3. No - control group improved (↓)  Provide Scores e.g. MD, p value etc |  |
| Alcohol use outcomes | 1. Yes - significant improvement compared with control (↑)  2. No significant difference between intervention and control (-) 3. No - control group improved (↓)  Provide Scores e.g. MD, p value etc |  |
| Sleep | 1. Yes - significant improvement compared with control (↑)  2. No significant difference between intervention and control (-) 3. No - control group improved (↓)  Provide Scores e.g. MD, p value etc |  |
| Other | 1. Yes - significant improvement compared with control (↑)  2. No significant difference between intervention and control (-) 3. No - control group improved (↓)  Provide Scores e.g. MD, p value etc |  |
