# Supplementary material for: Keep on truckin’: how effective are health behaviour interventions on truck drivers’ health? A systematic review and meta-analysis
Source: BMC Public Health. 2024 Sep 27;24:2623. doi: 10.1186/s12889-024-19929-1 (PMC11438120; doi:10.1186/s12889-024-19929-1)
Supplement: Supplementary file 4 — Supplementary Material 4. [file 12889_2024_19929_MOESM4_ESM.docx]

| Title | Authors | Year | Exclusion Reason |
| --- | --- | --- | --- |
| Work-life conflict among U.S. long-haul truck drivers: Influences of work organization, perceived job stress, sleep, and organizational support | Hege, A.; Lemke, M. K.; Apostolopoulos, Y.; Whitaker, B.; Sonmez, S. | 2019 | No intervention |
| Poor weight control, alcoholic beverage consumption and sudden sleep onset at the wheel among Italian truck drivers: A preliminary pilot study | Rosso, G. L.; Montomoli, C.; Candura, S. M. | 2016 | No intervention |
| Lifestyle, health aspects and work among truck drivers | Masson, V. A.; Monteiro, M. I. | 2010 | Wrong Language |
| Lifestyle counselling in promoting obese commercial drivers? Health ? Design and feasibility of a RCT | Puhkala, J.; Kukkonen-Harjula, K.; Hublin, C.; Karmeniemi, P.; Mansikkamaki, K.; Olkkonen, S.; Partinen, M.; Sallinen, M.; Aittasalo, M.; Fogelholm, M. | 2011 | Conference Abstract |
| Economic benefits of care management for OSA in a prospective cohort of professional truck drivers | Durmer, J. S.; Haigh, C.; Voien, D.; Kristjansson, S.; Thomas, D | 2014 | Conference Abstract |
| Physiotherapeutic intervention for reducing musculoskeletal pain and improving sleep quality in truck drivers working irregular shifts | Lemos L.C.; Moreno C.R.C. | 2022 | Conference Abstract |
| Adapting the workplace power men's weight loss program for long-distance truck drivers: physical activity and weight loss outcomes | Young, M.; Drew, R.; Morgan, P. | 2019 | Wrong intervention |
| Quality of online physical activity information for long-haul truck drivers | Gorczynski, P.; Patel, H. | 2014 | Wrong study design |
| Relationship of APAP adherence in commercial truck drivers at the initiation of treatment and after 270 days | Licata, C.; Nolte, C. M.; McWhirter, D. Y.; Bessler, M.; Eisenstadt, M. L. | 2011 | Conference Abstract |
| Shifting Gears: Physical inactivity, unhealthy diet choices and chronic disease risks in Australian truck drivers | Gilson, N.; Pavey, T.; Vandelanotte, C.; Duncan, M.; Wright, O.; Gomersall, S.; Trost, S.; Brown, W. | 2014 | Conference Abstract |
| Economic Impact of the BP DownShift Program on Blood Pressure Control Among Commercial Driver License Employees | Greene, Beth L.; Miller, Jeffrey D.; Brown, T Michelle; Harshman, Robert S.; Richerson, Gerald T.; Doyle, Joseph J. | 2009 | Wrong intervention |
| Program Evaluation of Fit to Pass, a Remotely Accessible Health Promotion Program for Commercial Motor Vehicle Truck Drivers | Snyder P.; Carbone E.; Heaton K.; Hammond S. | 2024 | Wrong study design |
| The Effect of Tailored Web-Based Feedback and Optional Telephone Coaching on Health Improvements: A Randomized Intervention Among Employees in the Transport Service Industry | Solenhill, M.; Grotta, A.; Pasquali, E.; Bakkman, L.; Bellocco, R.; Trolle Lagerros, Y | 2016 | Wrong population |
| Intervention of seat adjustment among drivers of forest tractors | Perkiö-Mäkelä, M.; Riihimäki, H. | 1997 | Wrong intervention |
| Developing measures of fatigue using an alcohol comparison to validate the effects of fatigue on performance | Williamson, A. M.; Feyer, A. M.; Mattick, R. P.; Friswell, R.; Finlay-Brown, S. | 2001 | Wrong intervention |
| Design, Development and Evaluation of Driver Wellness Programs: Technical Memorandum Number 3: Pilot Test Results and Marketing Plan | Roberts, S; York, J | 1999 | Wrong intervention |
| Case study of a healthy eating intervention for Swedish lorry drivers | Gill, P. E.; Wijk, K. | 2004 | Wrong outcomes |
| Truckies' nutrition and physical activity: A cross-sectional survey in Queensland, Australia | Sendall, M. C.; McCosker, L. K.; Ahmed, R.; Crane, P. | 2019 | No intervention |
| Weight loss intervention for professional truck drivers | Vash, P.; Graff, C. | 2014 | Conference Abstract |
| The structured health intervention for truckers (SHIFT) cluster randomised controlled trial: a mixed methods process evaluation | Guest A.J.; Paine N.J.; Chen Y.-L.; Chalkley A.; Munir F.; Edwardson C.L.; Gray L.J.; Johnson V.; Ruettger K.; Sayyah M.; Sherry A.; Troughton J.; Varela-Mato V.; Yates T.; King J.; Clemes S.A. | 2022 | Wrong outcomes |
| Job stress, fatigue, and job dissatisfaction in Dutch lorry drivers: Towards an occupation specific model of job demands and control | De Croon, E. M.; Blonk, R. W. B.; De Zwart, B. C. H.; Frings-Dresen, M. H. W.; Broersen, J. P. J. | 2002 | No intervention |
| Shifting gears: Patterns and changes in workday sedentary, stationary and movement time in Australian truck drivers | Gilson,; Pavey, T.; Gomersall, S.; Duncan, M.; Vandelanotte, C.; Wright, O.; Trost, S.; Brown, W. | 2015 | Conference abstract |
| Effects of lifestyle counselling on weight reduction and metabolic syndrome in obese male professional drivers - NCT00893646 | Puhkala, J.; Mansikkamaki, K.; Kukkonen-Harjula, K.; Aittasalo, M.; Tokola, K.; Hublin, C.; Karmeniemi, P.; Olkkonen, S.; Partinen, M.; Sallinen, M.; Fogelholm, M. | 2014 | Duplicate |
| Reducing Commercial Truck Driver BMI Through Motivational Interviewing and Self-Efficacy | Wilson, J. L.; Wolf, D. M.; Olszewski, K. A. | 2018 | Duplicate |
| Adapting the workplace power men's weight loss program for long-distance truck drivers: physical activity and weight loss outcomes | Young, M.; Drew, R.; Morgan, P. | 2019 | Conference Abstract |
| Nonadherence with employer-mandated sleep apnea treatment and increased risk of serious truck crashes | Burks, S. V.; Anderson, J. E.; Bombyk, M.; Haider, R.; Ganzhorn, D.; Jiao, X.; Lewis, C.; Lexvold, A.; Liu, H.; Ning, J.; Toll, A.; Hickman, J. S.; Mabry, E.; Berger, M.; Malhotra, A.; Czeisler, C. A.; Kales, S. N. | 2016 | Wrong intervention |
| Information technology and road transport industry: how does IT affect the lorry driver? | de Croon, Einar M.; Kuijer, P. Paul F. M.; Broersen, Jake P. J.; Frings-Dresen, Monique H. W. | 2004 | Wrong intervention |
| Louisiana trucking companies' implementation of workplace health promotion programs: Explanatory sequential mixed method case study | Houghtaling B.; Kourouma P.; Pradhananga N.; Balis L. | 2023 | No intervention |
| Random alcohol testing reduced alcohol-involved fatal crashes of drivers of large trucks | Snowden, C. B.; Miller, T. R.; Waehrer, G. M.; Spicer, R. S. | 2007 | No intervention |
| Impact of overnight traffic noise on sleep quality, sleepiness, and vigilant attention in long-haul truck drivers: Results of a pilot study | Popp, R. F.; Maier, S.; Rothe, S.; Zulley, J.; Cronlein, T.; Wetter, T. C.; Rupprecht, R.; Hajak, G. | 2015 | Wrong intervention |
| Shifting gears: Patterns and changes in workday sedentary, stationary and movement time in Australian truck drivers | Gilson,; Pavey, T.; Gomersall, S.; Duncan, M.; Vandelanotte, C.; Wright, O.; Trost, S.; Brown, W. | 2015 | Conference abstract |
| The impacts of rest breaks and stretching exercises on lower back pain among commercial truck drivers in Iran | Ghasemi, M.; Khoshakhlagh, A. H.; Ghanjal, A.; Yazdanirad, S.; Laal, F. | 2020 | Wrong outcomes |
| Evaluation of a prevention program for the truck transport | Gillet, P. | 2021 | Wrong language |
| Chronic disease risks and use of a smartphone application during a physical activity and dietary intervention in Australian truck drivers | Gilson, N. D.; Pavey, T. G.; Vandelanotte, C.; Duncan, M. J.; Gomersall, S. R.; Trost, S. G.; Brown, W. J. | 2016 | Duplicate |
| Weight Control Intervention for Truck Drivers: The SHIFT Randomized Controlled Trial, United States | Olson, R.; Wipfli, B.; Thompson, S. V.; Elliot, D. L.; Anger, W. K.; Bodner, T.; Hammer, L. B.; Perrin, N. A. | 2016 | Duplicate |
| Enhancing physical activity knowledge exchange strategies for Canadian long-haul truck drivers | Gorczynski, P. F.; Edmunds, S.; Lowry, R. | 2020 | No intervention |
| Stress, fatigue, health, and risk of road traffic accidents among professional drivers: the contribution of physical inactivity | Taylor, A. H.; Dorn, L. | 2006 | No intervention |
| Associations between high caffeine consumption, driving safety indicators, sleep and health behaviours in truck drivers | Filtness, A. J.; Hickman, J. S.; Mabry, J. E.; Glenn, L.; Mao, H.; Camden, M.; Hanowski, R. J. | 2020 | No intervention |
| Shifting gears: Process evaluation of an activity tracker and smart phone application to promote healthy lifestyle choices in Australian truck drivers | Gilson, N.; Pavey, T.; Gomersall, S.; Vandelanotte, C.; Duncan, M.; Wright, O.; Trost, S.; Brown, W. | 2014 | Conference abstract |
| An educational intervention to mitigate sleepiness in road transport | Sallinen, M.; Pylkkonen, M.; Hyvarinen, H. K.; Puttonen, S.; Sihvola, M. | 2014 | Conference abstract |
| Reducing Commercial Truck Driver BMI Through Motivational Interviewing and Self-Efficacy | Wilson, J. L.; Wolf, D. M.; Olszewski, K. A. | 2018 | Duplicate |
| Truckies and health promotion: A "hard-to-reach" group without a "proper" workplace | Hill, M. A.; Sendall, M. C.; McCosker, L. K. | 2015 | No intervention |
| Process Evaluation of a Mobile Weight Loss Intervention for Truck Drivers | Wipfli, B.; Hanson, G.; Anger, K.; Elliot, D. L.; Bodner, T.; Stevens, V.; Olson, R. | 2019 | Wrong outcomes |
| Alertness management strategies among long-haul truck drivers and airline pilots | Sallinen, M. | 2018 | Conference abstract |
| Case study of a healthy eating intervention for Swedish lorry drivers | Gill, P. E.; Wijk, K. | 2004 | Wrong intervention |
| A structured health intervention for truckers (SHIFT) | Johnson, V.; Troughton, J.; Varela Mato, V.; Clemes, S.; Davies, M. | 2020 | Conference abstract |
| Cluster randomised controlled trial to investigate the effectiveness and cost-effectiveness of a Structured Health Intervention for Truckers (the SHIFT study): A study protocol | Clemes, S. A.; Varela Mato, V.; Munir, F.; Edwardson, C. L.; Chen, Y. L.; Hamer, M.; Gray, L. J.; Bhupendra Jaicim, N.; Richardson, G.; Johnson, V.; Troughton, J.; Yates, T.; King, J. A | 2019 | No intervention |
| Truckers drive their own assessment for obstructive sleep apnea: A collaborative approach to online self-assessment for obstructive sleep apnea | Smith, B.; Phillips, B. A. | 2011 | Wrong study design |
| Process Evaluation of a Mobile Weight Loss Intervention for Truck Drivers | Wipfli, B.; Hanson, G.; Anger, K.; Elliot, D. L.; Bodner, T.; Stevens, V.; Olson, R. | 2019 | No intervention |
| The impacts of rest breaks and stretching exercises on lower back pain among commercial truck drivers in Iran | Ghasemi, M.; Khoshakhlagh, A. H.; Ghanjal, A.; Yazdanirad, S.; Laal, F. | 2018 | Duplicate |
| Alertness management strategies among long-haul truck drivers and airline pilots | Sallinen, M. | 2018 | Conference abstract |
| Effectiveness of health promotion programmes for truck drivers: A systematic review | Ng, M. K.; Yousuf, B.; Bigelowa, P. L.; Van Eerd, D. | 2015 | Wrong study design |
| A structured health intervention for truckers (SHIFT) | Johnson, V.; Troughton, J.; Varela Mato, V.; Clemes, S.; Davies, M. | 2020 | Conference abstract |
| Feedback actigraphy and sleep among long-haul truck drivers | Heaton, K. L.; Rayens, M. K. | 2010 | Duplicate |
| The impact of an m-Health financial incentives program on the physical activity and diet of Australian truck drivers | Gilson, N. D.; Pavey, T. G.; Wright, O. R.; Vandelanotte, C.; Duncan, M. J.; Gomersall, S.; Trost, S. G.; Brown, W. J. | 2017 | Duplicate |
| The use of active living every day to improve mass transit district employees' physical activity affect and enjoyment | Das, B. M.; Petruzzello, S. J. | 2015 | Wrong population |
| Shifting Gears: Physical inactivity, unhealthy diet choices and chronic disease risks in Australian truck drivers | Gilson, N.; Pavey, T.; Vandelanotte, C.; Duncan, M.; Wright, O.; Gomersall, S.; Trost, S.; Brown, W. | 2014 | Conference abstract |
| Weight loss intervention for professional truck drivers | Vash, P.; Graff, C. | 2014 | Conference abstract |
| Circadian Allertness Simulator for Fatigue Risk Assessment in Transportation: Application to Reduce Frequency and Severity of Truck Accidents | Moore-Ede, M.; Heitmann, A.; Guttkuhn, R.; Trutschel, U.; Aguirre, A.; Croke, D. | 2004 | Wrong study design |
| Circadian alertness simulator for fatigue risk assessment in transportation: application to reduce frequency and severity of truck accidents | Moore-Ede, M.; Heitmann, A.; Guttkuhn, R.; Trutschel, U.; Aguirre, A.; Croke, D. | 2004 | Duplicate |
| Time in nature associated with decreased fatigue in UK truck drivers | Longman, D. P.; Shaw, C. N.; Varela-Mato, V.; Sherry, A. P.; Ruettger, K.; Sayyah, M.; Guest, A.; Chen, Y. L.; Paine, N. J.; King, J. A.; Clemes, S. A. | 2021 | No intervention |
| Shifting gears: Process evaluation of an activity tracker and smart phone application to promote healthy lifestyle choices in Australian truck drivers | Gilson, N.; Pavey, T.; Gomersall, S.; Vandelanotte, C.; Duncan, M.; Wright, O.; Trost, S.; Brown, W. | 2014 | Conference abstract |
| Lone workers attitudes towards their health: views of Ontario truck drivers and their managers | McDonough, B.; Howard, M.; Angeles, R.; Dolovich, L.; Marzanek-Lefebvre, F.; Riva, J. J.; Laryea, S. | 2014 | Wrong study design |
| The Obesogenic Environment of Commercial Trucking: A Worksite Environmental Audit and Implications for Systems-Based Interventions | Apostolopoulos, Y.; Lemke, M.; Sonmez, S.; Hege, A. | 2016 | No intervention |
| Lorry driver's cardiovascular health, physical activity and sedentary behaviours-phase 1 of the shift study NIHR Leicester-Lough borough Diet, Lifestyle and Physical Activity Biomedical Research Unit | Veronica Varela Mato, V.; O'Shea, O.; King, J.; Yates, T.; Stensel, D. J.; Biddle, S. J.; Clemes, S. A. | 2016 | No intervention |
| Comparison between two programmes for reducing the levels of risk indicators of heart diseases among male professional driver | Hedberg, G. E.; Wikstrom-Frisen, L.; Janlert, U. | 1998 | Deuplicate |
| Truckies and health promotion: Using the ANGELO framework to understand the workplace's role | Sendall, M. C.; Crane, P.; McCosker, L.; Fleming, M.; Biggs, H. C.; Rowland, B. | 2017 | No intervention |
| A process evaluation of tobacco-related outcomes from a telephone and print-delivered intervention for motor freight workers | Quintiliani, L.; Yang, M.; Sorensen, G. | 2010 | Duplicate |
| A new health promotion model for lone workers: Results of the safety & health involvement for truckers (SHIFT) pilot study | Olson, R.; Anger, W. K.; Elliot, D. L.; Wipfli, B.; Gray, M. | 2009 | Duplicate |
| Biomechanical investigation of prolonged driving in an ergonomically designed truck seat prototype | Cardoso, M.; McKinnon, C.; Viggiani, D.; Johnson, M. J.; Callaghan, J. P.; Albert, W. J. | 2018 | Wrong intervention |
| Effect of mobile testing, treatment and care management for obstructive sleep apnea on adherence and clinical outcomes in professional drivers over 12 months | Durmer, J. S.; Haigh, C.; Voien, D.; Kristjansson, S.; Thomas, D. | 2014 | Conference abstract |
| The impacts of rest breaks and stretching exercises on lower back pain among commercial truck drivers in Iran | Ghasemi, M.; Khoshakhlagh, A. H.; Ghanjal, A.; Yazdanirad, S.; Laal, F. | 2020 | Duplicate |
| Sleep and Safety Decision-Making Among Truck Drivers | Heaton, K.; Mumbower, R.; Childs, G. | 2021 | Wrong study design |
| Risks endemic to long-haul trucking in North America: strategies to protect and promote driver well-being | Apostolopoulos, Y.; Lemke, M.; Sonmez, S. | 2014 | No intervention |
| Associations between Musculoskeletal Conditions Risk, Sedentary Behavior, Sleep, and Markers of Mental Health: A Cross-Sectional Observational Study in Heavy Goods Vehicle Drivers. Musculoskeletal Conditions Risk in HGV Drivers | Varela-Mato, V.; Clemes, S. A.; King, J.; Munir, F. | 2019 | No intervention |
| An educational intervention to mitigate sleepiness in road transport | Sallinen, M.; Pylkkonen, M.; Hyvarinen, H. K.; Puttonen, S.; Sihvola, M. | 2014 | Conference abstract |
| Diet associations with sleep and fatigue among truck drivers: Baseline results from the shift randomized controlled trial | Thompson, S.; Hohn, E.; Wipfli, B.; Olson, R. | 2015 | Conference abstract |
