# Supplementary material for: Keep on truckin’: how effective are health behaviour interventions on truck drivers’ health? A systematic review and meta-analysis
Source: BMC Public Health. 2024 Sep 27;24:2623. doi: 10.1186/s12889-024-19929-1 (PMC11438120; doi:10.1186/s12889-024-19929-1)

Supplementary File 6: Meta-analyses of change in outcomes between pre-and post-intervention for total cholesterol, blood pressure, and fasting blood glucose.


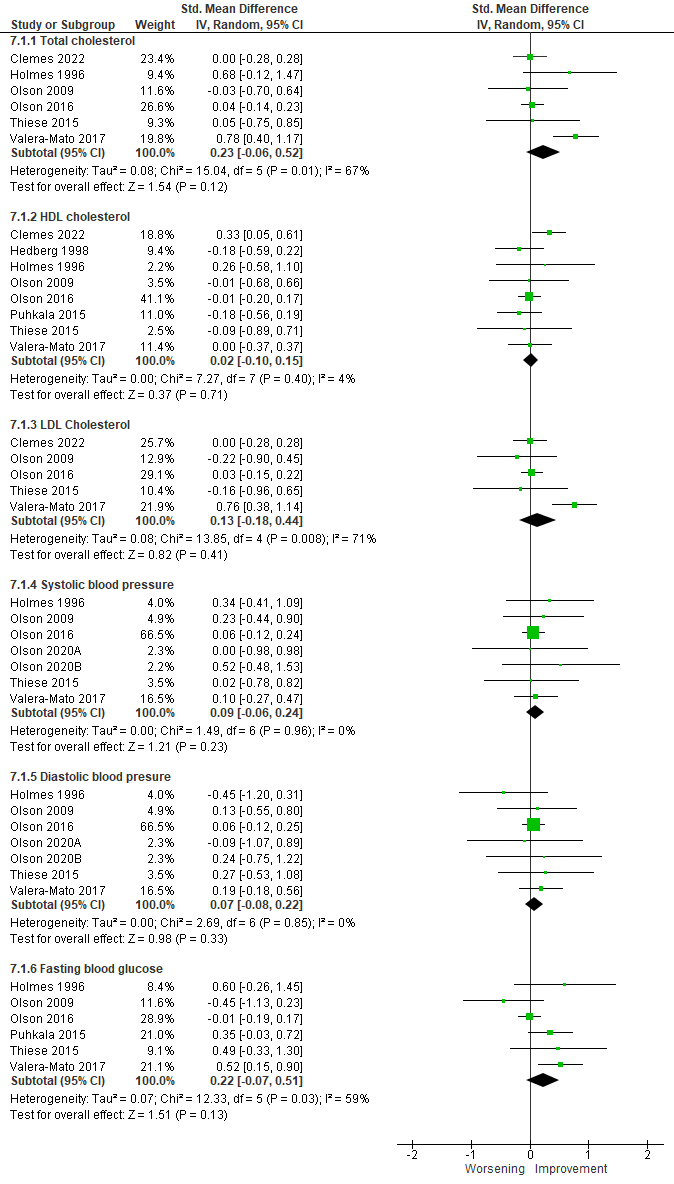

Supplement: Supplementary file 6 — Supplementary Material 6. [file 12889_2024_19929_MOESM6_ESM.docx]
